# Associations of family income with cognition and brain structure in USA children: prevention implications

**DOI:** 10.1038/s41380-021-01130-0

**Published:** 2021-05-14

**Authors:** Dardo Tomasi, Nora D. Volkow

**Affiliations:** 1 National Institute on Alcohol Abuse and Alcoholism, Bethesda, MD USA; 2 National Institute on Drug Abuse, Bethesda, MD USA

**Keywords:** Neuroscience, Predictive markers

## Abstract

Poverty, as assessed by several socioeconomic (SES) factors, has been linked to worse cognitive performance and reduced cortical brain volumes in children. However, the relative contributions of the various SES factors on brain development and the mediating effects between cognition and brain morphometry have not been investigated. Here we used cross-sectional data from the ABCD Study to evaluate associations among various SES and demographic factors, brain morphometrics, and cognition and their reproducibility in two independent subsamples of 3892 children. Among the SES factors, family income (FI) best explained individual differences in cognitive test scores (stronger for crystallized than for fluid cognition), cortical volume (CV), and thickness (CT). Other SES factors that showed significant associations with cognition and brain morphometrics included parental education and neighborhood deprivation, but when controlling for FI, their effect sizes were negligible and their regional brain patterns were not reproducible. Mediation analyses showed that cognitive scores, which we used as surrogate markers of the children’s level of cognitive stimulation, partially mediated the association of FI and CT, whereas the mediations of brain morphometrics on the association of FI and cognition were not significant. These results suggest that lack of supportive/educational stimulation in children from low-income families might drive the reduced CV and CT. Thus, strategies to enhance parental supportive stimulation and the quality of education for children in low-income families could help counteract the negative effects of poverty on children’s brain development.

## Introduction

Despite being a high-income country, 16% of children in the US live below the poverty line [1], and in low- and middle-income countries up to 43% of children younger than 5 years (250 million) might not reach their developmental potential due to poverty [2]. Children living in poverty are exposed to increased risks (including poor health and education, malnutrition, and unstimulating home atmospheres that influence brain maturation), perform poorly in school, have lower educational attainment [3], and frequently show symptoms of psychopathology [4]. Low socioeconomic status (SES) in childhood/adolescence can have profound consequences in adult social behaviors, cognitive abilities, and health [5] given the plasticity of executive functions during the early years of life. Socioeconomic disadvantage has been linked with cognitive deficits [6] and impaired socio-emotional development [7], and frequently manifest as disease conditions later in life [8].

Only recently, with the advent of large repositories of magnetic resonance imaging (MRI) datasets, researchers have begun to investigate the relatively small effects (*η*^2^ < 0.1) of SES on brain structure [9, 10]. For instance, family income (FI) and parental education (PED), two traditional measures of childhood SES that correlate with one another, were significantly associated with the thickness of the prefrontal cortex (PFC) in children and adolescents [11]. The effect of SES on cortical surface area was found to be particularly prominent in frontoparietal regions supporting language, spatial skills, and executive functions [12]. Beyond PED and FI, risk of lead exposure (RLE), which is more frequent among the poor [13], has been associated with lower intelligence [14], and a recent study showed that higher RLE, as estimated from residential data, was linked to lower cognitive scores and increasingly smaller cortical surface areas and brain volumes in children from low-income but not in those from high-income families [15]. Excess weight (EW) in children, which in the US is more prevalent among those with lower SES [16], was also associated with lower executive function and lower cortical thickness in PFC areas [17].

Numerous studies have studied the influence of SES on life outcomes, and related their effects on mental health and cognition through their influence on the brain [18], and several studies have also documented that the distal effects of SES on the brain are mediated by environmental factors (i.e., “proximal factors”) such as stress, linguistics, cognitive stimulation, parenting practices, prenatal care, toxins, sleep, or nutrition [9]. Previous studies have also documented the importance of parental support in brain development [19–21]. For example, children who were adopted when they were older had smaller prefrontal volumes than those who were adopted when they were younger, indicating that the longer the duration of childhood deprivation the worse the outcomes [22]. In another study, young adults who lived their first years of life (3–41 months) in orphanages under very deprived environments and were subsequently adopted, showed smaller total brain volumes (8.6% smaller) than non-deprived adoptees despite the intervening stimulation provided by their adoptee families [23].

Other relevant factors influenced by SES that affect brain development include recreational activities such as time spent on passive or interactive screen media activity (SMA) [24, 25], family composition, and interactions (e.g., number of siblings, SIB, biological parents, and adults living with the child) [26, 27], and neighborhood deprivation [15, 28]. Thus in our analyses, we included SMA considering that 97% of US children have at least one electronic item in their bedrooms [29], SIB considering that the number of only-child families in the Adolescent Brain Cognitive Development (ABCD) Study is relatively high (67%) and neighborhood deprivation. The associations between factors that are influenced by SES and brain structure in children suggests that there are multiple variables contributing to poverty’s negative effects on cognition and on brain development. However, the relative contribution of various SES factors on cognition and brain morphometrics has not been comprehensively assessed. While multiple studies have reported on the mediation of brain morphometrics in cognition, the mediation of cognition, which we used as surrogate for levels of child cognitive stimulation, on the relationship between SES and brain morphometrics has not been evaluated. Further, the reproducibility of the effects of SES on brain measures in children has not been investigated nor have confounds from intra-scan head motion [30, 31] always been properly controlled [32].

The present study aims to quantify the relative contribution of various socioeconomic [FI, RLE, PED, and area deprivation index (ADI)], family environment (SIB, SMA), and demographic (EW, gender, and age) factors on cognition and brain morphometrics (CV and CT), and their reproducibility in 7784 children from the ABCD Study. We strictly controlled for scanner manufacturer (SM), head motion, intracranial volume (ICV), and race, using factorial analysis of covariance (ANCOVA) and causal mediation analysis (CMA). Our working hypothesis was that compared to other SES indicators, FI would have the strongest effects on cognition and brain development, and that after covarying for FI the effects of the other SES factors on cognition and brain morphometrics would be significantly reduced. We also hypothesized that proximal factors such as educational achievement, extracurricular activities, sleep, BMI, and/or pubertal hormones would mediate the effects of FI on brain morphometrics.

## Materials and methods

### Participants

The ABCD Study is a 10-year longitudinal study involving 21 data collection sites across the United States [33]. Centralized institutional review board (IRB) approval was obtained from the University of California, San Diego IRB. Study sites obtained approval from their local IRBs. Written, informed consent was provided by each parent. Children were fluent in English and provided written assent for their participation. All ethical regulations were complied with during data collection and analysis. Recruitment closely represented demographic variables (sex, race, ethnicity, parental marital status and education, and income) of the general US population [34]. Children were excluded if they had contraindications for MRI, intellectual, medical, or neurological issues, or poor English-language proficiency [35].

The 2019 ABCD 2.0 data release [36] includes baseline data for more than 11,800 children. To control for intra-scan head motion, in this study we included data (Table S1) from 10,712 children with available mean framewise displacement (FD) data corresponding to resting-state fMRI. A participant’s data were additionally excluded if brain segmentation did not pass ABCD quality control (QC) (*N* = 384), or demonstrated moderate or severe head motion (*N* = 992); miss sex (*N* = 1, defined at birth), age (*N* = 0), race/ethnicity (*N* = 14), weight or height (*N* = 21), FI bracket (*N* = 876), PED (*N* = 345), ADI: median FI (ADI, *N* = 640), or the cognitive total composite score from the NIH Toolbox (*N* = 354); or was an outlier for body mass index (BMI > 50; *N* = 6). Thus, a total of 2928 children were excluded, 705 of which meet more than one exclusion criterion. For the variables of interest (Supplementary Table 1), there were complete data for 7784 children, which were randomly split into Discovery and Validation samples of equal size (*N* = 3892) to assess the reproducibility of the results (Table 1). In addition, an independent group of 262 children (Normality sample) with missing PED but otherwise complete data was identified among excluded children in the ABCD dataset to perform tests of normality on the morphometric data.

**Table 1:** Characteristics of the Discovery and Validation ABCD samples.

	Included			Excluded
	Discovery	Validation	*P* val	
Family income	7.2 ± 2.4	7.3 ± 2.3	n.s.	5.1 ± 3.9^a^
Average neighborhood income ($)	76,641 ± 34,369	77,416 ± 35,181	n.s.	58,712 ± 46,203^a^
Risk of lead exposure	4.9 ± 3.1	4.9 ± 3.1	n.s.	4.4 ± 3.6^a^
Excess weight (lean/overweight)	2530/1362	2522/1370	n.s.^b^	1825/1103^a^
Siblings (yes/non)	1280/2612	1271/2621	n.s.^b^	1981/947^a^
Screen media activity (h/week)	20.8 ± 16.9	20.4 ± 16.9	n.s.	22.3 ± 18.6^a^
Parental education level	16.6 ± 2.6	16.7 ± 2.5	n.s.	14.0 ± 5.8^a^
Sex (male/female)	2042/1850	2044/1848	n.s.^b^	1507/1421
Age (months)	118 ± 8	119 ± 8	n.s.	118 ± 7
Intracranial volume (L)	1.52 ± 0.15	1.52 ± 0.15	n.s.	1.49 ± 0.15^a^
Race (White/African American/Hispanic/Asian/Other^c^)	2149/486/788/61/408	2189/503/743/68/389	n.s.^b^	1311/570/639/91/317^a^
Scanner manufacturer (GE/Phillips/Siemens)	792/417/2683	814/450/2628	n.s.^b^	911/485/1532^a^
Mean framewise displacement (mm)	0.26 ± 0.26	0.27 ± 0.27	n.s.	0.36 ± 0.34^a^
Mean cortical thickness (mm)	2.80 ± 0.09	2.80 ± 0.09	n.s.	2.75 ± 0.12^a^
Total cortical volume (mL)	601 ± 58	601 ± 56	n.s.	589 ± 59^a^
Total cortical area (mm^2^)	187 ± 18	187 ± 18	n.s.	187 ± 20
Fluid composite	96 ± 17	97 ± 17	n.s.	83 ± 34^a^
Crystallized composite	106 ± 18	107 ± 18	n.s.	94 ± 36^a^
Cognitive total composite	101 ± 17	102 ± 18	n.s.	87 ± 36^a^

^a^Significant difference between included and excluded participants (*P* < 0.05).

^b^*χ*^2^-test.

^c^More than one race; *P* val: statistical differences between the Discovery and Validation samples.

### Residential history data

Fifteen additional ADI dimensions (education, household disparity, median home, rent and mortgage values, percentages of homeowners, families living in poverty and crowdedness, unemployment, singles, homes without car and telephone, and population density) and three uniform crime reports (total crime, DUI, and drug abuse) were extracted from residential history-derived scores to assess neighborhood deprivation and safety.

### Behavioral data

We used the uncorrected standard fluid, crystallized and total cognition composite scores, which were calculated within the NIH Toolbox [37]. The Fluid Composite scores were calculated using the following tests: (1) pattern comparison processing speed; (2) list-sorting working memory; (3) picture sequence memory; (4) Flanker; and (5) the dimensional change card sort. The crystallized composite scores were calculated using (6) the oral reading recognition and (7) the picture vocabulary tests. The fluid and crystallized composites were used to calculate the total cognition composite scores.

### Morphometric data

We used measures of CV, and CT, which were estimated from T1-weighted scans. The MRI data acquisition procedures and image processing analysis of the ABCD study are described in detail elsewhere [38, 39]. In brief, T1w and T2w structural scans with 1-mm isotropic resolution were collected using adult-size multi-channel coils, and harmonized image acquisition protocols for 3Tesla Siemens, Phillips, and General Electric scanners at 21 sites. During MRI, children restfully watched a child-friendly movie in the scanner [38]. Structural scans were collected using real-time motion detection and correction [38]. QC procedures were based on automated mean and SD of brain values [39]. In addition, trained raters inspected T1w and T2w images for poor quality, artifacts such as motion-related ghosting, blurring, or ringing that prevent brain segmentation [39]. T1w and T2w images were corrected for scanner-specific gradient distortions. Intensity inhomogeneity was corrected using a B1-bias field, and image intensity was harmonized across participants. Cortical and subcortical segmentation of T1w images was computed with FreeSurfer [39], which has been validated for use in children [40]. We used 148 cortical ROIs automatically segmented according to surface-based nonlinear registration to an atlas of cortical folding patterns [41]. Trained raters reviewed the accuracy of the segmentation and the artifacts of the cortical surface reconstruction, indicating if motion, intensity inhomogeneity, white matter underestimation, pial overestimation, and magnetic susceptibility artifacts were either absent, mild, moderate, or severe, and gave on overall QC score for the cortical surface reconstruction [39].

### Additional data

The numbers of biological parents and adults living with the child were additionally used to assess family composition, and school grades and sleep hours were used to assess educational achievement and sleep behavior, for all children (*N* = 7784). In ABCD subsamples with available data, we separately assessed children’s access to alcohol (*N* = 3405) and cigarettes (*N* = 1238), as measures of parental oversight, extracurricular activities [sports (*N* = 2342), arts (*N* = 1927), reading (*N* = 2261), and music listening (*N* = 2229)], as measures of enrichment opportunities, and sex hormone levels [estradiol, HSE (*N* = 1177), testosterone, ERT (*N* = 2707), and dehydroepiandrosterone, DHEA (*N* = 2811)] as measures of pubertal development.

### Statistical analyses

We first tested the normal distributions of total CV and its regression slopes for the continuous variables (see text below) using the Shapiro–Wilk normality test [42] and the Normality sample (*W* > 0.99; *P* > 0.05). Then, a factorial ANCOVA was conducted in R to study main effects of FI [ten income brackets: (1) <$5000; 2) $5000–12,000; (3) $12,000–16,000; (4) $16,000–25,000; (5) $25,000–35,000; (6) $35,000–50,000; (7) $50,000–75,000; (8) $75,000–100,000; (9) $100,000–200,000; (10) >$200,000] on the dependent variable Y, which represents either brain morphometrics (CV and CT) or the total cognition composite, while controlling for differences in sex, age, ICV, and race [White, African American, Hispanic, Asian, Other], which were used as covariates of no interest. Since head motion is also a concern for pediatric structural and functional neuroimaging [30, 31, 43], we also controlled for the subjects’ tendency to move their head while resting in the scanner, as informed by the subjects’ average FD during 5-min resting-state fMRI scans, using FD as an additional covariate of no interest. Because the ABCD morphometric measures vary significantly with SM [39], when modeling morphometrics we used SM (GE, Philips, Siemens) as an additional covariate of no interest.

Socioeconomic (SES) variables [FI, RLE (US census tract [15]), PED (the average educational level achieved by the parent; 22 levels), SMA (number of weekly hours the child spends watching TV shows, movies, or videos; playing video games; texting; video chatting; or visiting social network sites), and ADI] were highly correlated with one another (0.61 > |*R*| > 0.13; *P* < 2E − 16), sharing a significant fraction of the variance.

We used Akaike (AIC) and Bayesian (BIC) information criteria to select the SES variable providing the better fit to the data. Specifically, we contrasted AIC and BIC values for five different models summarized by

1 $${Y}\sim {Z} + {\mathrm{EW}} + {\mathrm{IB}} + {\mathrm{Covariates}}$$

where EW (L: underweight or lean, O: overweight or obese) and SIB (N: no siblings, Y: one or more siblings) are categorical factors and *Z* stands for FI (model 1), ADI (model 2), PED (model 3), RLE (model 4) or SMA (model 5). BMI was calculated in kg/m^2^ from the participant’s weight and height, and overweight-obese (underweight-lead) was defined as BMI > (<) 85th percentile for children and teens of the same age and sex. Since these comparisons demonstrated that model 1 provided the best fit to morphometrics and cognition composites, a full model:

2 $${Y}\sim {\mathrm{FI}} + {\mathrm{ADI}} + {\mathrm{PED}} + {\mathrm{RLE}} + {\mathrm{SMA}} + {\mathrm{EW}} + {\mathrm{SIB}} + {\mathrm{Covariates}}$$

was additionally tested to assess residual effects of secondary SES variables (ADI, PED, RLE, SMA) relative to that of the main SES variable (FI), and to assess regional effects of FI, EW, and SIB on brain morphometrics. Partial *η*^2^ was used in conjunction with ANCOVA to estimate effect sizes of categorical and continuous factors [42]. Tukey’s “Honest Significant Difference” method [44] was used in conjunction with ANCOVA to compute confidence intervals on the differences between the means of the levels of a categorical factor. Bonferroni corrections for multiple comparisons were based on 148 ROIs.

### Principal component analysis (PCA) and hierarchical clustering

PCA, conducted with the stats v3.6.2 R-package, was used for dimensionality reduction and exploratory data analysis. The hierarchy of clusters was visualized as a heatmap with a dendrogram.

### Causal mediation analysis (CMA)

The “mediation” package [45] and a global model including all factors in Eq. (2) were used to estimate causal mediation effects with continuous and discrete mediators [46]. One thousand bootstrapping samples and a heteroskedasticity-consistent estimator for the covariance matrix were used to estimate the average direct (ADE) and causal mediation (ACME) effects.

## Results

**Fig. 1:**
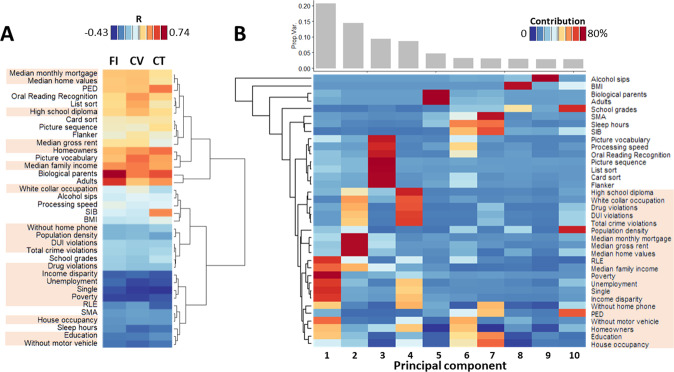
Demographic, cognitive, family SES, and health behavior variables. **A** Scaled heatmap with hierarchical clustering showing the correlations of these variables with family income (FI), cortical volume (CV), and thickness (CT). **B** Bar plot showing that the top ten principal components captured 72% of the variance (top) and a scaled heatmap with hierarchical clustering showing relative contributions of the principal components for each of the variables (bottom). Highlighted variable labels correspond to residential history-derived scores.

All 36 demographic, cognitive family SES, and health behavior variables had significant correlations with FI, CV, and CT (0.03 < *R* < 0.60, Fig. 1A). Ten principal components (PC) accounted for 72% of the variance in SES and cognitive measures (Fig. 1B). Poverty indicators (ADI) predominated in PC# 1, 2, and 4, which accounted for 44% of the variance; cognitive measures predominated in PC# 3, which accounted for 9% of the variance.

### Socioeconomic factors and cognition

**Fig. 2:**
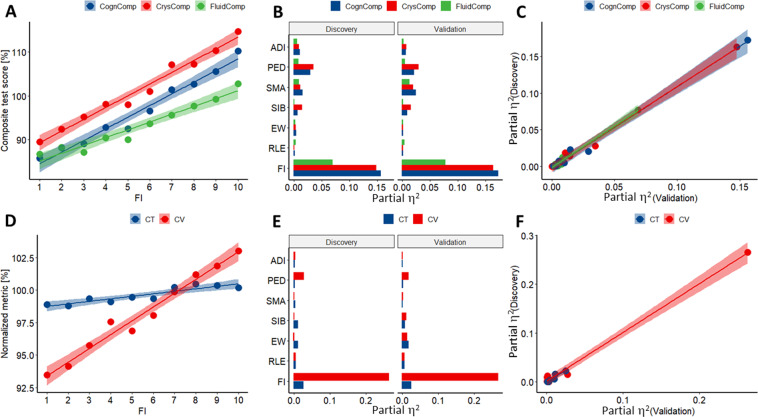
Demographics and morphometrics variables versus socioeconomic factors. Linear associations of family income (FI) with fluid (FluidComp), crystallized (CrysComp), and total (CognComp) cognition composites (**A**), and with relative measures of total cortical volume (CV) and mean cortical thickness (CT) (**D**), averaged within participants of the same FI bracket. Effect size (partial *η*^2^) corresponding to nine ANCOVA factors and two independent samples (Discovery and Validation) for three cognitive scores (**B**) and three morphometrics (**E**). Scatter plots showing the reproducibility of the effect sizes (**C,** **F**). FI brackets: (1) <$5000; (2) $5000–12,000; (3) $12,000–16,000; (4) $16,000–25,000; (5) $25,000–35,000; (6) $35,000–50,000; (7) $50,000–50,000; (8) $75,000–100,000; (9) $100,000–200,000; (10) >$200,000. Factorial ANCOVA with nine factors of interest [FI, RLE, excess weight (EW), siblings (SIB), SMA, PED, sex, age, and area deprivation index (ADI)], and four covariates of no interest (race, intracranial volume, scanner manufacturer, and intra-scan head motion). Discovery and Validation samples of equal size (*N* = 3892), matched by demographic, socioeconomic, morphometric, and cognitive variables (Table 1).

Fluid, crystallized, and total cognitive test composites were positively correlated with FI (Fig. 2A), ADI, and PED, factors that were strongly correlated across participants (*R* > 0.42, *N* = 7,784, *P* < 2E − 16), and worsened with increased RLE and longer SMA (Fig. S1), which were negatively associated with FI (*R* < −0.28, *N* = 7784, *P* < 2E − 16). Regression slopes for these factors were reproducible and steeper for crystallized than for fluid cognitive scores (*F*_1,16_ > 39.0, *P* < 1E − 05, ANCOVA; Fig. 2A). The effect sizes on cognition were larger for FI (0.069 < *η*^2^ < 0.156, medium-large effect size) than for SMA (0.010 < *η*^2^ < 0.015, small effect size) and PED (0.015 < *η*^2^ < 0.035, small effect size; for crystallized and total composites, not for fluid) and altogether explained ~20% of the variance in cognition (Fig. 2B) and were reproducible (Fig. 2C). The AIC applied to five different ANCOVA models to determine which SES factor best-fitted the cognition composite corroborated that FI had the best fit (ΔAIC = AIC − AIC_FI_ > 38.5) (Table S2). After accounting for FI, the residual effects of ADI, PED, RLE, SMA, and siblings (SIB) on cognition were significant and reproducible, but for EW were not reproducible and for RLE and ADI were not significant (Tables S2 and S3).

### Socioeconomic factors and brain morphometry

**Fig. 3:**
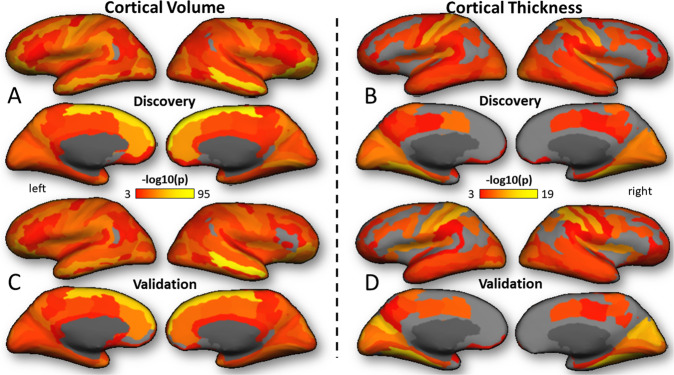
Regional effects of family income (FI) on cortical volume and thickness. Cortical renderings of statistical significance for the effect of FI on brain morphometrics showing the pattern of the effect in the Discovery (**A,** **B**) and Validation (**C,** **D**) samples. Factorial ANCOVA with nine factors of interest (FI, risk of lead exposure, excess weight, siblings, screen media activity, parental education, sex, age, and area deprivation index), and four covariates of no interest (race, intracranial volume, scanner manufacturer, and intra-scan head motion).

Total CV and average CT had positive correlation with FI, PED, and ADI, and negative correlation with RLE and SMA (Figs. 1D and S1A and Tables S4 and S5), paralleling the effects of FI on cognition, and the regression slopes were steeper for CV than for CT (*F*_1,16_ > 80.4, *P* < 1E − 07, ANCOVA). As for cognition, we estimated the effects of the SES factors on morphometrics using five different ANCOVA models (Table S4) and found that FI had the best fit (AIC was lower for FI than for other SES factors) (ΔAIC > 2.8). The stronger correlations between FI and CV were in superior frontal, middle temporal, orbital and precentral gyri, and anterior cingulum (Fig. 3A), whereas for CT they were in sensory cortices, posterior default-mode network regions, and language areas (*P* < 1E − 18; Fig. 3B). The effects of FI on CV and CT were highly reproducible (Figs. 3 and S2 and Table S6).

The residual effects of PED and SIB on brain morphometrics were reproducible (Table S4). FI had large reproducible effects on CV (*η*^2^ > 0.195) and smaller reproducible effects in CT (*η*^2^ = 0.024), whereas the other variables showed small but reproducible effects (0.010 < *η*^2^ < 0.030) on total CV (SIB and PED), and CT (EW). Children with siblings had smaller cortical area (*F*_1,7765_ = 153.4, *P* < 2E − 16; Fig. S1), also resembling the effects of SIB on cognition (see Supplementary Results) but had thicker cortex than children without siblings [<1.5%; TukeyHSD test; Fig. S2]. Overweight/obese children had thinner cortex than lean/underweight children [*F*_1,16_ = 5.0, *P* = 0.04, ANCOVA; Fig. S2].

### Mediation analysis

**Fig. 4:**
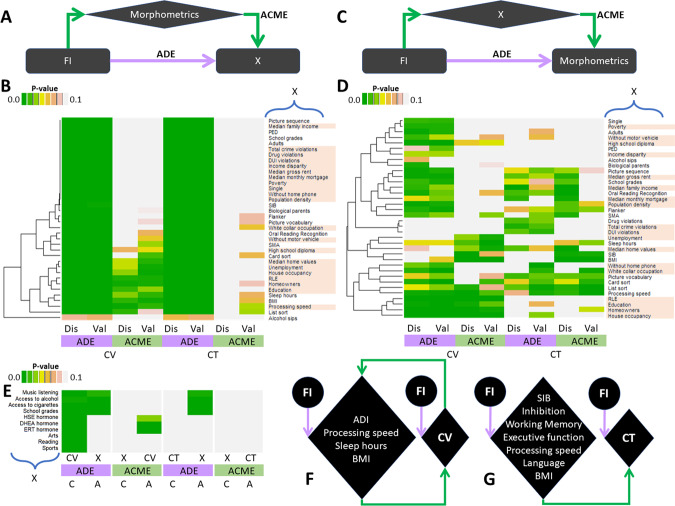
Causal mediation analysis (CMA). Mediation models (**A**, **C**) and unscaled heatmaps with hierarchical clustering (**B,** **D**) for average direct (ADE) and causal mediation (ACME) effects of cortical volume (CV) and thickness (CT) on the relationships between family income (FI) and 36 demographic, socioeconomic, and health behavior variables (X; **A**, **B**) and for those of X on the relationships between FI and the morphometrics (**C**, **D**) for Discovery (Dis) and Validation (Val) samples of 3892 children each. Separate CMA for selected subsamples assessing ADE and ACME of access to alcohol (*N* = 3405) and cigarettes (*N* = 1238), extracurricular sports (*N* = 2,342), arts (*N* = 1,927), reading (*N* = 2,261), and music listening (*N* = 2229), as well as pubertal estradiol, HSE (*N* = 1,177), testosterone, ERT (*N* = 2707) and dehydroepiandrosterone, DHEA (*N* = 2,811) hormones (X) on the relationships between FI and morphometrics, as well as those of CV and CT on the relationships between FI and X (**E**); the reproducibility of these pathways was not tested given the reduced size of these subsamples. Schematics highlighting reproducible ADE and ACME for CV (**F**) and CT (**G**). ADI area deprivation index, SIB siblings, PED parental education, BMI body mass index, RLE risk of lead exposure, SMA screen media activity. Highlighted variable labels correspond to residential history-derived scores.

CMA (Fig. 4A–D, F, G) demonstrated direct effects of FI on all demographic, cognitive, family SES, and health behavior variables, except alcohol sips (*P*_ADE_ < 2E − 16), as well as reproducible partial mediation effects of ADI (education, RLE, median home values, and homeowners, house occupancy and unemployment rates), sleep hours, BMI, and processing speed on the relationship between FI and CV (*P*_ACME_ < 0.05), and of inhibitory (Flanker), language (picture vocabulary), memory (card sort and list sort) and information processing, BMI, and the number of siblings on the relationship between FI and CT (*P*_ACME_ < 0.05).

### Pubertal hormones

CMA also demonstrated significant mediation effects of pubertal hormones (ERT and DHEA) on the relationship between FI and CV (*P*_ACME_ < 2E − 16; Fig. 4E). ERT and DHEA had significant negative correlation with FI (*P* = 1E − 04).

While CMA demonstrated direct effects of FI on school grades, music listening, and children’s access to alcohol and tobacco, it did not show significant mediation effects of these variables to CV or CT.

## Discussion

Here, studying the relative contribution of various SES factors on cognition (fluid and crystallized) and brain morphometrics in two independent ABCD samples of children, we corroborated our hypothesis that FI had the strongest associations with cognition and brain morphometrics.

FI had a reproducible contribution to inter-individual variations in cognitive test scores (partial *η*^2^ > 0.15), and to total CV (partial *η*^2^ > 0.20) and had a smaller though significant and reproducible association with CT (partial *η*^2^ < 0.03). Unique contributions from other factors, which correlated with FI (residual effects of PED, RLE, EW, SMA, and ADI) were significant, but their effect sizes were much smaller than for FI and accounted for only a small fraction of the variance in cognitive scores and in total CV (partial *η*^2^ < 0.03). Similar findings were reported by a prior study in 1099 typically developing 3–20 years old, which also showed that among the SES factors investigated, FI had the largest influence on brain structure [12].

FI had strong linear associations with the cognitive composites, consistent with prior studies [3, 6], which were steeper for crystallized than for fluid scores. This suggests that language abilities might be particularly vulnerable to growing up in poverty, presumably from lack of access to high-quality education as well as exposure to more complex verbal and written language during everyday family life. FI was also reproducibly associated with the fluent composite with medium effect size, which suggests that the ability to solve problems, think, and reason abstractly might be impaired in children from low-income families, presumably due to limited exposure to an environment that can promote the development of such skills. In parallel, we observed an association between FI and CV, particularly in superior frontal, middle temporal, orbital and precentral gyri, and anterior cingulate, and between FI and CT, particularly in sensory regions, precuneus and language areas.

The slope of the association between FI and CT (0.2% per income bracket) was less steep than for total CV (1% per income bracket), both in the Discovery and Validation samples, suggesting a weaker influence of FI for CT than for CV. Reduced CV in low-income family children could result from decreased gyrification during brain maturation [47], and the smaller effects on CT could reflect accelerated developmental thinning of the cortex [47]. The association of FI with CT was most prominent in sensory, default-mode, and language regions, a pattern remarkably similar to the autonomic brain network implicated in processing signals from the peripheral nervous system, personality, and emotions [48]. Thus, greater reactivity of the autonomic system in poor children might have accelerated pruning in these regions [49].

Higher education, better jobs, higher income, and better neighborhoods usually tend to go together, and though highly correlated [50] might have unique consequences on children’s brain development [51]. We found that higher PED (degree, or school grade/level completed by parents) was uniquely associated with better cognition scores and increased CV, independent of FI and other covariates in a reproducible way. However, the association of PED with average CT was not significant, consistent with prior studies [12]. These differences could reflect the fact that CV and CT, capture different evolutionary, genetic, and cellular factors [52, 53]. We also found that after accounting for FI, the residual effect of ADI was weakly associated with the cognition composites but did not show associations with any of the brain morphometrics.

In our study we assumed that cognitive performance is an indirect surrogate of the level of stimulation a child is exposed to and hypothesized that it would partially mediate effects of FI on brain morphometrics. Our findings corroborated this hypothesis and showed that scores on language and executive functions, including inhibitory-control and working memory, partially mediated the association of FI with CT, and those of processing speed partially mediated the effects of FI on both, CT and CV, consistent with the influence of family SES on children’s cognitive abilities [54]. These suggest that income-related cognitive stimulation (e.g., childcare quality, school quality, access to tutors and home learning environments, etc.) could have influenced the association between FI and children’s CV and CT. Prevention studies that have evaluated the effects of training parents on family management including problem-solving and support for academic activities were shown to prevent the adverse effects of poverty on brain development [55]. Unfortunately, the ABCD study has limited information of childcare data during early childhood development, so we cannot assess its modulation of FI effects on brain morphometrics. Also, we did not find mediation effects on the relationships between FI and brain morphometrics with two other surrogate markers of stimulation (children’s school grades and extracurricular activities). However, it should be noted that school grades in the ABCD dataset are currently not normalized to school’s rankings across the country, and the data on extracurricular activities is only available for 25% of the ABCD sample.

We found reproducible mediation effects of increased BMI on the association between lower FI and smaller CV and CT. The observed negative correlation between BMI and FI is consistent with the increase of BMI in children from poor neighborhoods [56]. Since higher BMI has been associated with lower brain volumes [57], our findings suggest that the associations between FI and CV and CT partially reflect higher BMI in children from low-income families. We also found reproducible mediation effects of sleep hours on the association between lower FI and smaller CV. Sleep is important for several brain functions as well as for the clearance of accumulating toxins from the brain [58]. The negative correlation between FI and sleep hours suggests that insufficient sleep may have contributed to smaller CV in children from low-income families. Similarly, in a subsample of *N* > 2700 ABCD children we found an intriguing mediation effect of pubertal hormones (ERT and DHEA) on the association between FI and CV. Since gonadal steroids levels increase during puberty and adolescence [59], the observed negative correlation between FI and pubertal hormones suggests delayed puberty in children from low-income families, which could be consistent with delayed neurocognitive maturation in lower-income environments [60]. Therefore, the mediation of the hormonal levels suggests that delayed puberty may have contributed to smaller CV in children from low-income families. However, note that other studies have reported an opposite association; that is accelerated puberty in girls from low SES [61]; this conflicting results might reflect characteristics of the ABCD sample such as lower representation of children of families with very low SES than prior studies.

Though the cross-sectional nature of baseline ABCD Study’s data does not allow us to confer causality, our findings in the context of the existing literature have public health implications that highlight the importance of strategies to minimize the adverse effects of poverty in children. Moreover, such preventive strategies have been shown not only to be beneficial to the children who were targeted but to have transgenerational effects improving cognition and mental health in their children when they become parents [62]. Further, the protective effects of prevention interventions against poverty reduced the poverty status of children when they reached young adulthood [63].

Additional limitations to our study include the narrow age range of participants, which limits the generalizability to other brain development stages. The ABCD sample’s representativenes of the US population is only partial. Specifically, while the ABCD sample and the general US population have similar PED at the lower levels (e.g., 68% of parents in ABCD and 62–67% of adults in US completed at least some college studies), a larger fraction completed the Batchelor’s degree in ABCD (55%) than in the US population (46%). The ABCD study has also relatively lower representation from families of very low incomes and this might have contributed to the discrepant findings we observed for the assocation between low FI and puberty. Also the number of only-child families in the ABCD study is relatively high (67%), and family environment may be radically different for only-child and multi-children families in terms of the children’s cognition, personality and affect characteristics [26], which is why we assessed the influence of SIB. However, the association with sibling might have differed in a population that had higher representation from families with very low incomes. Finally, the recently reported low reliability of the NIH-Toolbox cognitive battery [64], which will require further re-assesement, might limit the robustness of findings pertaining to cognition.

Here we show reproducible moderate associations of FI with cognition and brain structure. The mediation analyses suggest that lower cognition, insufficient sleep, EW, and crowded family environments in children raised in economically disadvantaged families might contribute to these disparities.

## Supplementary information

Supplementary material

## Data Availability

ABCD data are publicly available through the National Institute of Mental Health Data Archive (https://data-archive.nimh.nih.gov/abcd).
