# Supplementary material for: Associations of family income with cognition and brain structure in USA children: prevention implications
Source: Mol Psychiatry. 2021 May 14;26(11):6619–29. doi: 10.1038/s41380-021-01130-0 (PMC8590701; doi:10.1038/s41380-021-01130-0)
Supplement: Supplementary file 1 — Supplementary material [file 41380_2021_1130_MOESM1_ESM.docx]

Supporting Information for:

**Associations of Family Income with Cognition and Brain Structure in USA Children: Prevention Implications**

Dardo Tomasi*^1^ and Nora D. Volkow^1,2^

^1^National Institute on Alcohol Abuse and Alcoholism, Bethesda, MD, 20892

^2^National Institute on Drug Abuse, Bethesda, MD, 20892

**Supplementary results**

**Socioeconomic factors and cognition. To** investigate whether socioeconomic (SES) factors explained a significant fraction of the cognitive variability in the ABCD study we used cognitive scores from fluid, crystallized and total cognitive tests, measured with the National Institutes of Health (NIH) Toolbox [1] Cognitive composite scores improved significantly with family income (FI), area deprivation index (ADI), and parental education (PED), factors that were strongly correlated across participants (R>0.42, N=7,784, P<2E-16), and worsened with increased risk of lead exposure (RLE) and longer screen media activity (SMA; Fig S1A-C), which were negatively associated with FI (R<-0.28, N=7,784, P<2E-16). Differences in regression slopes for FI/PED between crystallized and fluid test composites were significant (F_1,16_>39.0, P<1E-05, ANCOVA) such that the regression slopes were steeper for crystallized than for fluid composite scores. To determine which of the highly intercorrelated SES factors (FI, ADI, PED, RLE, and SMA) best fitted the total cognition composite, we used 5 different ANCOVA models (Table S2), and Akaike’s (AIC) information criterion, which was lower for FI than for ADI, PED, RLE and SMA (ΔAIC=AIC − AIC_FI_>38.5). Next, we estimated the effect of FI as the main SES factor and the residual effects of ADI, PED, RLE, SMA as well as those of siblings (SIB) and excess weight (EW) on the cognition composites using the full ANCOVA model (Table S3). Children with siblings (SIB) had lower crystallized and total cognition composites than children without them [ΔCrysComp: 4.0, CI=(3.2,4.7); ΔCognComp: 2.6, CI=(1.9,3.3); Tukey’s Honest Significant Difference (HSD) test; S1D]. The effect of FI and SIB, and the residual effects of SMA, and PED on cognition were reproducible in the Discovery and Validation samples (Table S2). The effect of excess weight (EW) on the cognitive composites was significant only in the Discovery sample. The residual effects of RLE and ADI on cognitive scores were not significant.


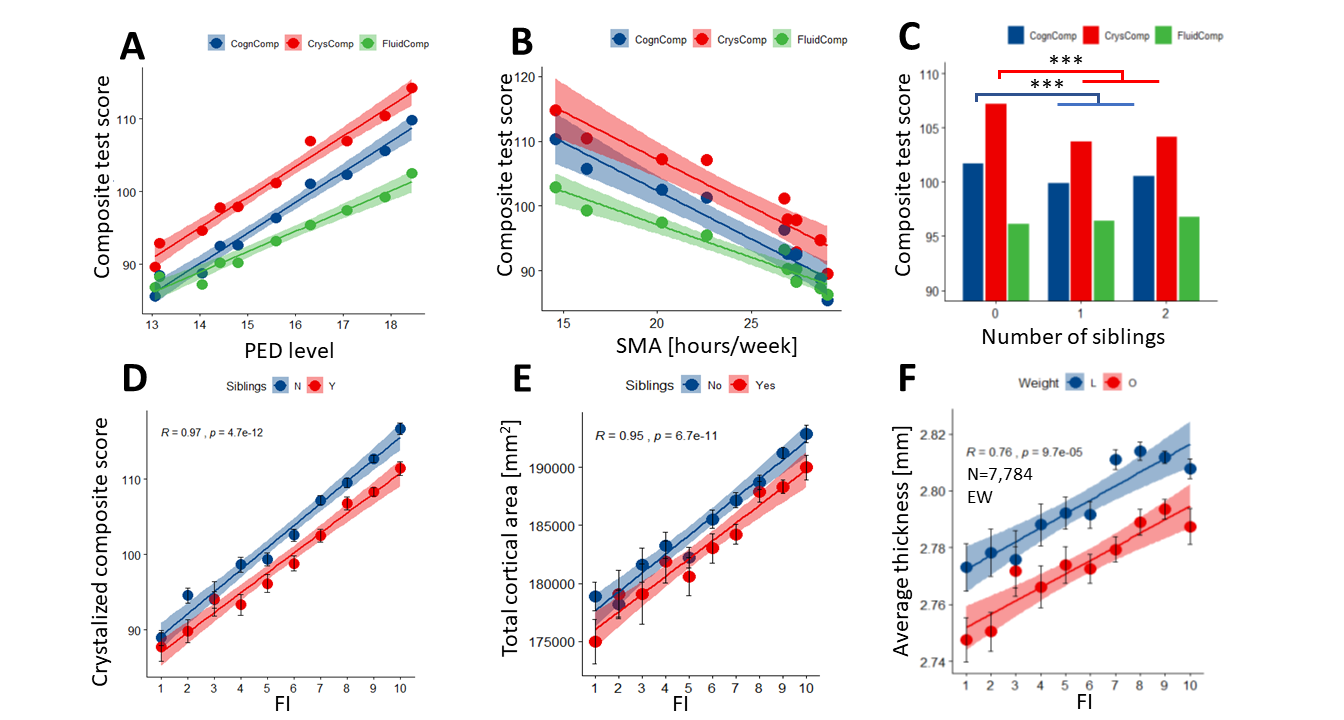


**Fig S1: Cognitive composites versus socioeconomic factors.** Linear associations of screen media activity (SMA, **A**) and parental education (PED, **B**) with fluid (FluidComp), crystallized (CrysComp), and total (CognComp), cognition composites averaged within participants of the same FI bracket. **C**) Bar plots showing that the crystallized and total cognitive composite scores were lower for children with siblings. Children with siblings (Y; red) had higher crystalized composite scores (**D**) and larger cortical area than those without them (N, red; **E**), and overweight/obese (O) children had thinner cortex than lean/underweight (L, blue) children (**H**)FI brackets: 1) < $5,000; 2) $5,000–12,000; 3) $12,000–16,000; 4) $16,000–25,000; 5) $25,000–35,000; 6) $35,000–50,000; 7) $50,000–50,000; 8) $75,000–100,000; 9) $100,000–200,000; 10) > $200,000. Factorial ANCOVA with 9 factors of interest [FI, risk of lead exposure (RLE), EW, SIB, SMA, PED, sex, age, and area deprivation index (ADI)], and 3 covariates of no interest (race, intracranial volume, and intra scan head motion). Discovery and Validation samples of equal size (N=3,892), matched by demographic, socioeconomic, morphometric and cognitive variables (Table 1).


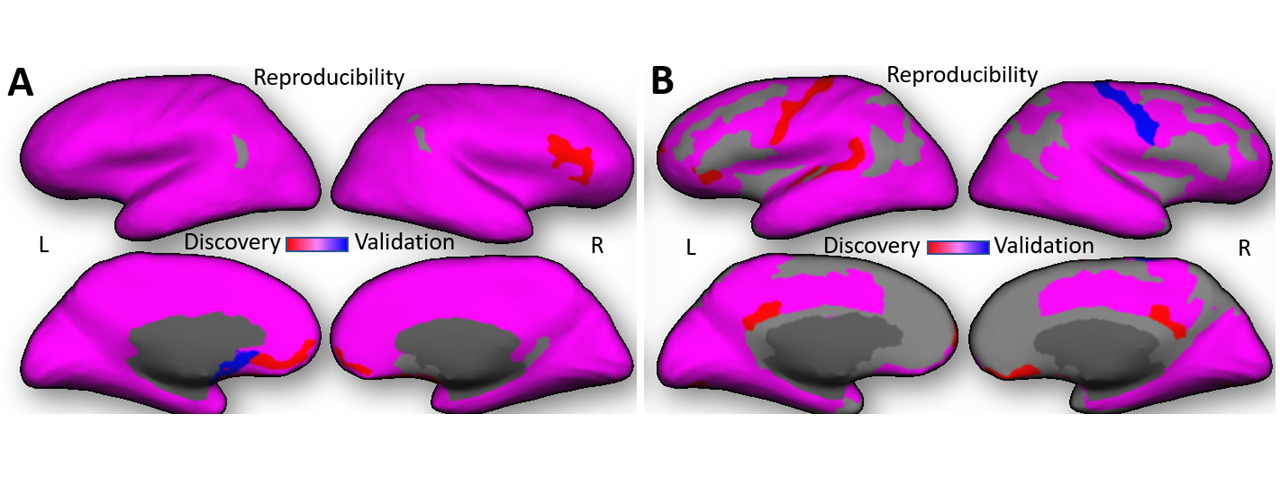


**Fig S2: Regional effects of family income (FI) on cortical volume and thickness.** Cortical renderings of the overlap (violet) of significant effects of FI on cortical volume (**A**) and cortical thickness (**B**) in the Discovery (red) and Validation (blue) samples. Factorial ANCOVA with 9 factors of interest (FI, risk of lead exposure, excess weight, siblings, screen media activity, parental education, sex, age, and area deprivation index), and 4 covariates of no interest (race, intracranial volume, scanner manufacturer and intra scan head motion). Discovery and Validation samples of equal size (N=3,892), matched by demographic, socioeconomic, morphometric and cognitive variables (Table 1).

**Regional effects of excess weight (EW) and siblings (SIB).** We conducted similar post hoc ROI analyses to assess the regional patterns for the effects of EW and SIB on cortical thickness. In the Discovery sample, overweight/obese children demonstrated 1.4±0.7 % thinner cortex in frontal, temporal, and occipital areas, compared to lean/underweight children that were strongest in bilateral anterior cingulate cortex and medial orbitofrontal cortex (Fig S2A and S2B; Table S6), and that except for the central and orbital lateral sulci were reproduced in the Validation sample (Fig S2C). FI and EW demonstrated reproducible small effect sizes (1.5-2.5%) on average cortical thickness within these regions, but other independent variables in the factorial ANCOVA did not have significant effect size (η^2^<0.01; Fig S2D). The EW x FI interaction effect on cortical thickness was not significant. In the Discovery sample, children with siblings demonstrated 0.7±0.5 % thicker cortex in frontal, temporal, parietal, and occipital areas, than children without siblings that was strongest in left anterior cingulate (Fig S2E and S2F; Table S6) and reproduced in the Validation sample (Fig S2G; Table S6). The SIB x FI interaction effect on cortical thickness was not significant (F_1,16_=2.7, P=0.12, ANCOVA). SIB and FI demonstrated reproducible small effect sizes (1.0-2.5%) on cortical thickness within these regions, but other independent variables in the factorial ANCOVA did not have a significant effect size (η^2^<0.01; Fig S2H).


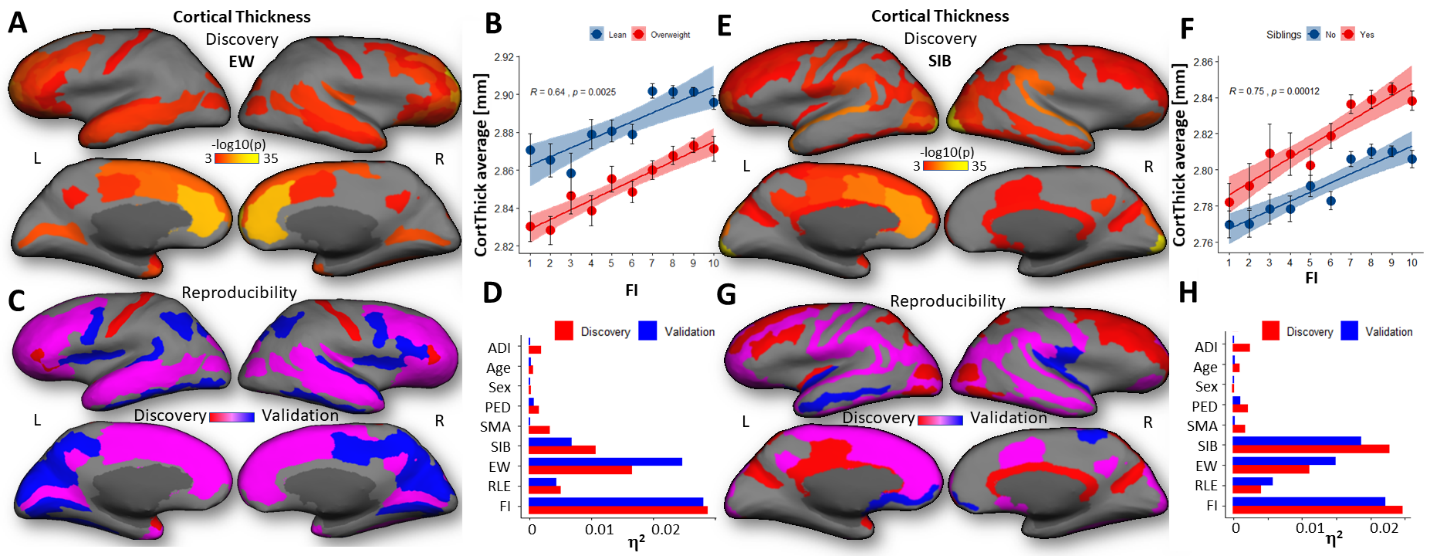


**Fig S2: Regional effects of excess weight (EW) and siblings (SIB) on cortical thickness.** Cortical renderings of statistical significance for the effect of EW and SIB on brain morphometrics showing the pattern of the effect in the Discovery sample (**A** and **E**) as well as the overlap of the patterns (violet) in the Discovery (red) and Validation (blue) samples (**C**, and **G**). Scatter plots show the linear effects of family income (FI) on cortical thickness (CortThick) for lean/underweight (Lean) and for overweight/obese (Overweight) children (**B**), as well as for children with (Yes) or without (No) siblings (**F**). Bar plots show the effect sizes (partial η^2^) for all 9 factors of interest for the Discovery and validation samples (**G**). Factorial ANCOVA model controlling for race, intracranial volume, scanner manufacturer and intra scan head motion. Effects surviving Bonferroni corrections for 148 comparisons. Error bars are standard errors. RLE: risk of lead exposure; SMA: screen media activity; PED: parental education; ADI: area deprivation index. Discovery and Validation matched samples of equal size (N=3,892).

Table S1: Data tables and variables (NDA Release 2.0/2.0.1)

| Dataset | Description |
| --- | --- |
| Demographics (pdem02) | |
| demo_comb_income | Total combined family income for the past 12 months |
| demo_prnt_ed | Caregiver (Parent) 1 Education Level |
| demo_prtnr_ed | Caregiver (Parent) 2 Education Level |
| fam_roster_2c-15c | Relationship of people living at the address of the child |
| NIH Toolbox (abcd_tbss01) | |
| nihtbx_fluidcomp_uncorrected | Cognition Fluid Composite Uncorrected Standard Score |
| nihtbx_cryst_uncorrected | Crystallized Composite Uncorrected Standard Score |
| nihtbx_totalcomp_uncorrected | Cognition Total Composite Score Uncorrected Standard Score |
| nihtbx_picvocab_uncorrected | Picture Vocabulary Test Age 3+ v2.0 Uncorrected Standard Score |
| nihtbx_flanker_uncorrected | Flanker Inhibitory Control and Attention Test Ages 8-11 v2.0 Uncorrected Standard Score |
| nihtbx_list_uncorrected | List Sorting Working Memory Test Age 7+ v2.0 Uncorrected Standard Score |
| nihtbx_cardsort_uncorrected | Dimensional Change Card Sort Test Ages 8-11 v2.0 Uncorrected Standard Score |
| nihtbx_pattern_uncorrected | Pattern Comparison Processing Speed Test Age 7+ v2.0 Uncorrected Standard Score |
| nihtbx_picture_uncorrected | Picture Sequence Memory Test Age 8+ Form A v2.0 Uncorrected Standard Score |
| nihtbx_reading_uncorrected | Oral Reading Recognition Test Age 3+ v2.0 Uncorrected Standard Score |
| Structural MRI (abcd_smrip201 or abcd_mrisdp101) | |
| smri_vol_scs_intracranialv | Intra cranial volume in mm^3^ |
| mrisdp_1 to mrisdp_151 | Mean cortical thickness in mm |
| mrisdp_303 to mrisdp_453 | Mean cortical area in mm^2^ |
| mrisdp_454 to mrisdp_604 | Mean cortical volume in mm^3^ |
| Residential History (abcd_rhds01) | |
| reshist_addr1_valid | Validity of Primary Residential Address |
| reshist_addr1_leadrisk | Estimated lead risk in census tract of primary residential address |
| reshist_addr1_adi_income | Area Deprivation Index (ADI): Median family income |
| reshist_addr1_p1vlnt | Uniform Crime Reports (UCR): adult violent crimes |
| reshist_addr1_drugtot | UCR: drug abuse violations total |
| reshist_addr1_dui | UCR: DUI |
| reshist_addr1_adi_edu_l | ADI: Percentage of population aged >=25 y with <9 y of education |
| reshist_addr1_adi_edu_h | ADI: Percentage of population aged >=25 y with high school diploma |
| reshist_addr1_adi_work_c | ADI: Percentage of employed persons aged >=16 y in white collar occupations |
| reshist_addr1_adi_in_dis | ADI: Income disparity defined by Singh |
| reshist_addr1_adi_home_v | ADI: Median home value |
| reshist_addr1_adi_rent | ADI: Median gross rent |
| reshist_addr1_adi_mortg | ADI: Median monthly mortgage |
| reshist_addr1_adi_home_o | ADI: Percentage of owner |
| reshist_addr1_adi_crowd | ADI: Percentage of occupied housing units with >1 person per room (crowding) |
| reshist_addr1_adi_unemp | ADI: Percentage of civilian labor force population aged >=16 y unemployed |
| reshist_addr1_adi_pov | ADI: Percentage of families below the poverty level |
| reshist_addr1_adi_sp | ADI: Percentage of single |
| reshist_addr1_adi_ncar | ADI: Percentage of occupied housing units without a motor vehicle |
| reshist_addr1_adi_ntel | ADI: Percentage of occupied housing units without a telephone |
| reshist_addr1_popdensity | UN adjusted population density |
| Longitudinal Tracking (abcd_lt01) | |
| site_id_l Site | ID Number |
| American Community Survey Post Stratification Weights (acspsw03) | |
| interview_age | Age |
| gender | Sex |
| race_ethnicity | Race/Ethnicity |
| rel_family_id | Family ID Number |
| rel_relationship | Number of siblings |
| Youth Screen Time Survey (abcd_stq01) | |
| screen1_wkdy_y to screen12_wkdy_y | Number of hours spent on screen media activity |
| Youth Anthropometrics Modified From PhenX (abcd_ant01) | |
| anthroweightcalc | Average Measured Weight |
| anthroheightcalc | Standing Height Average |
| FreeSurfer QC (freesqc01) | |
| fsqc_qu_motion | Motion score |
| fsqc_qc | QC score |
| rsfMRI Gordon Network Correlations (abcd_betnet02) | |
| rsfmri_c_ngd_meanmotion | Average framewise displacement in mm |
| MRI Info (abcd_mri01) | |
| mri_info_manufacturer | Imaging device manufacturer |
| Youth Substance Use Interview (abcd_ysu02) | |
| su_isip_1_calc | Lifetime number of alcohol sips |
| Parent Community Risk and Protective Factors (CRPF; abcd_crpf01) | |
| su_risk_p_1 | Access to beer, wine, or hard liquor |
| su_risk_p_2 | Access to cigarettes |
| su_risk_p_3 | Access to e-cigarettes |
| Sports and Activities Involvement Questionnaire (SAIQ; abcd_ysu02) | |
| sai_p_dance_nmonth_l | Months per year the child participated in dance |
| dance_perwk_l | Days per week the child participated in dance |
| sai_p_dance_tspent_l | Approximate duration of the dance session |
| sai_p_base_nmonth_l | Months per year the child participated in baseball/Softball |
| base_perwk_l | Days per week the child participated in baseball/Softball |
| sai_p_base_tspent_l | Approximate duration of the baseball/Softball session |
| sai_p_basket_nmonth_l | Months per year the child participated in basketball |
| basket_perwk_l | Days per week the child participated in basketball |
| sai_p_basket_tspent_l | Approximate duration of the basketball session |
| sai_p_climb_nmonth_l | Months per year the child participated in climbing |
| climb_perwk_l | Days per week the child participated in climbing |
| sai_p_climb_tspent_l | Approximate duration of the climbing session |
| sai_p_fhock_nmonth_l | Months per year the child participated in field hokey |
| fhock_perwk_l | Days per week the child participated in field hokey |
| sai_p_fhock_tspent_l | Approximate duration of the field hokey session |
| sai_p_fball_nmonth_l | Months per year the child participated in football |
| fball_perwk_l | Days per week the child participated in football |
| sai_p_fball_tspent_l | Approximate duration of the football session |
| sai_p_gym_nmonth_l | Months per year the child participated in gymnastics |
| gym_perwk_l | Days per week the child participated in gymnastics |
| sai_p_gym_tspent_l | Approximate duration of the gymnastics session |
| sai_p_ihock_nmonth_l | Months per year the child participated in ice hockey |
| ihock_perwk_l | Days per week the child participated in ice hockey |
| sai_p_ihock_tspent_l | Approximate duration of the ice hockey session |
| sai_p_polo_nmonth_l | Months per year the child participated in horseback riding |
| polo_perwk_l | Days per week the child participated in horseback riding |
| sai_p_polo_tspent_l | Approximate duration of the ice horseback riding session |
| sai_p_iskate_nmonth_l | Months per year the child participated in skating |
| iskate_perwk_l | Days per week the child participated in skating |
| sai_p_iskate_tspent_l | Approximate duration of the skating session |
| sai_p_m_arts_nmonth_l | Months per year the child participated in martial arts |
| m_arts_perwk_l | Days per week the child participated in martial arts |
| sai_p_m_arts_tspent_l | Approximate duration of the martial arts session |
| sai_p_lax_nmonth_l | Months per year the child participated in lacrosse |
| lax_perwk_l | Days per week the child participated in lacrosse |
| sai_p_lax_tspent_l | Approximate duration of the lacrosse session |
| sai_p_rugby_nmonth_l | Months per year the child participated in rugby |
| rugby_perwk_l | Days per week the child participated in rugby |
| sai_p_rugby_tspent_l | Approximate duration of the rugby session |
| sai_p_skate_nmonth_l | Months per year the child participated in skateboarding |
| skate_perwk_l | Days per week the child participated in skateboarding |
| sai_p_skate_tspent_l | Approximate duration of the skateboarding session |
| sai_p_sboard_nmonth_l | Months per year the child participated in skiing/snowboarding |
| sboard_perwk_l | Days per week the child participated in skiing/snowboarding |
| sai_p_sboard_tspent_l | Approximate duration of the skiing/snowboarding session |
| sai_p_soc_nmonth_l | Months per year the child participated in soccer |
| soc_perwk_l | Days per week the child participated in soccer |
| sai_p_soc_tspent_l | Approximate duration of the soccer session |
| sai_p_surf_nmonth_l | Months per year the child participated in surfing |
| surf_perwk_l | Days per week the child participated in surfing |
| sai_p_surf_tspent_l | Approximate duration of the surfing session |
| sai_p_wpolo_nmonth_l | Months per year the child participated in swimming |
| wpolo_perwk_l | Days per week the child participated in swimming |
| sai_p_wpolo_tspent_l | Approximate duration of the swimming session |
| sai_p_tennis_nmonth_l | Months per year the child participated in tennis |
| tennis_perwk_l | Days per week the child participated in tennis |
| sai_p_tennis_tspent_l | Approximate duration of the tennis session |
| sai_p_run_nmonth_l | Months per year the child participated in track/running |
| run_perwk_l | Days per week the child participated in track/running |
| sai_p_run_tspent_l | Approximate duration of the track/running session |
| sai_p_mma_nmonth_l | Months per year the child participated in mixed martial arts |
| mma_perwk_l | Days per week the child participated in mixed martial arts |
| sai_p_mma_tspent_l | Approximate duration of the mixed martial arts session |
| sai_p_vball_nmonth_l | Months per year the child participated in volleyball |
| vball_perwk_l | Days per week the child participated in volleyball |
| sai_p_vball_tspent_l | Approximate duration of the volleyball session |
| sai_p_yoga_nmonth_l | Months per year the child participated in yoga |
| yoga_perwk_l | Days per week the child participated in yoga |
| sai_p_yoga_tspent_l | Approximate duration of the yoga session |
| sai_p_music_nmonth_l | Months per year the child played a music instrument |
| music_perwk_l | Days per week the child played a music instrument |
| sai_p_music_tspent_l | Approximate duration of the music session |
| sai_p_art_nmonth_l | Months per year the child participated in drawing/painting |
| art_perwk_l | Days per week the child participated in drawing/painting |
| sai_p_art_tspent_l | Approximate duration of the drawing/painting session |
| sai_p_drama_nmonth_l | Months per year the child participated in theater/acting |
| drama_perwk_l | Days per week the child participated in theater/acting |
| sai_p_drama_tspent_l | Approximate duration of the theater/acting session |
| sai_p_crafts_nmonth_l | Months per year the child participated in crafts like knitting |
| crafts_perwk_l | Days per week the child participated in crafts |
| sai_p_crafts_tspent_l | Approximate duration of the crafts session |
| sai_p_chess_nmonth_l | Months per year the child participated in competitive games |
| chess_perwk_l | Days per week the child participated in competitive games |
| sai_p_chess_tspent_l | Approximate duration of the game session |
| sai_p_collect_nmonth_l | Months per year the child participated in hobbies |
| collect_perwk_l | Days per week the child participated in hobbies |
| sai_p_collect_tspent_l | Approximate duration of the hobby’s session |
| Parent Sleep Disturbance Scale for Children (SDS; abcd_sds01) | |
| sleepdisturb1_p | Number of sleep hours per night on most nights |
| Hormone Saliva Salimetric Scores (abcd_hsss01) | |
| hormone_scr_dhea_mean | Salimetrics hormone test DHEA mean of measures (pg/mL) |
| hormone_scr_hse_mean | Salimetrics hormone test estradiol (HSE) mean of measures (pg/mL) |
| hormone_scr_ert_mean | Salimetrics hormone test testosterone (ERT) mean of measures (pg/mL) |

Table S2: Statistical significance corresponding to 5 different ANCOVA models associating Crystalized, fluid, and total cognition composites with family income (FI; model 1), area deprivation index (ADI; model 2), parental education (PED; model3), risk of lead exposure (RLE; model 4), and screen media activity (SMA; model 5). Excess weight (EW) and siblings (SIB) were categorical factors of all 5 models

| Composite | Sample | P-value | | | F-value | | | Partial η^2^ | | |
| --- | --- | --- | --- | --- | --- | --- | --- | --- | --- | --- |
|  | **Model 1** | **FI** | **EW** | **SIB** | **FI** | **EW** | **SIB** | **FI** | **EW** | **SIB** |
| Crystalized | Discovery | 2.3E-131 | 1.0E-03 | 2.1E-13 | 6.4E+02 | 1.1E+01 | 5.4E+01 | 1.4E-01 | n.s. | 1.4E-02 |
|  | Validation | 1.1E-145 | n.s. | 3.1E-13 | 7.2E+02 | 3.3E+00 | 5.4E+01 | 1.6E-01 | n.s. | 1.4E-02 |
| Fluid | Discovery | 5.9E-61 | 1.6E-03 | n.s. | 2.8E+02 | 9.9E+00 | 9.6E-02 | 6.8E-02 | n.s. | n.s. |
|  | Validation | 1.9E-68 | n.s. | n.s. | 3.2E+02 | 2.2E+00 | 4.4E-01 | 7.6E-02 | n.s. | n.s. |
| Total | Discovery | 1.2E-139 | 4.5E-05 | 9.0E-06 | 6.9E+02 | 1.7E+01 | 2.0E+01 | 1.5E-01 | n.s. | n.s. |
|  | Validation | 1.2E-155 | 4.4E-02 | 5.9E-07 | 7.7E+02 | 4.0E+00 | 2.5E+01 | 1.7E-01 | n.s. | n.s. |
|  |  |  |  |  |  |  |  |  |  |  |
|  | **Model 2** | **ADI** | **EW** | **SIB** | **ADI** | **EW** | **SIB** | **ADI** | **EW** | **SIB** |
| Crystalized | Discovery | 9.7E-89 | 8.5E-07 | 2.9E-11 | 4.2E+02 | 2.4E+01 | 4.5E+01 | 9.8E-02 | n.s. | 1.1E-02 |
|  | Validation | 4.0E-89 | 1.2E-03 | 8.3E-10 | 4.2E+02 | 1.1E+01 | 3.8E+01 | 9.8E-02 | n.s. | 1.0E-02 |
| Fluid | Discovery | 3.9E-48 | 4.9E-05 | n.s. | 2.2E+02 | 1.7E+01 | 1.7E-03 | 5.3E-02 | n.s. | 4.4E-07 |
|  | Validation | 2.4E-43 | 1.5E-02 | n.s. | 2.0E+02 | 5.9E+00 | 2.4E-04 | 4.8E-02 | n.s. | n.s. |
| Total | Discovery | 7.4E-101 | 1.7E-08 | 1.5E-04 | 4.8E+02 | 3.2E+01 | 1.4E+01 | 1.1E-01 | n.s. | n.s. |
|  | Validation | 7.6E-97 | 5.1E-04 | 1.2E-04 | 4.6E+02 | 1.2E+01 | 1.5E+01 | 1.1E-01 | n.s. | n.s. |
|  |  |  |  |  |  |  |  |  |  |  |
|  | **Model 3** | **PED** | **EW** | **SIB** | **PED** | **EW** | **SIB** | **PED** | **EW** | **SIB** |
| Crystalized | Discovery | 2.2E-132 | 4.7E-04 | 1.2E-10 | 6.5E+02 | 1.2E+01 | 4.2E+01 | 1.4E-01 | n.s. | 1.1E-02 |
|  | Validation | 3.9E-125 | 4.3E-03 | 1.8E-08 | 6.1E+02 | 8.2E+00 | 3.2E+01 | 1.4E-01 | n.s. | 1.0E-02 |
| Fluid | Discovery | 1.5E-51 | 3.9E-04 | n.s. | 2.4E+02 | 1.3E+01 | 1.2E-01 | 5.7E-02 | n.s. | n.s. |
|  | Validation | 5.0E-43 | 1.2E-02 | n.s. | 1.9E+02 | 6.3E+00 | 2.7E-01 | 4.8E-02 | n.s. | n.s. |
| Total | Discovery | 1.3E-131 | 7.8E-06 | 5.6E-04 | 6.4E+02 | 2.0E+01 | 1.2E+01 | 1.4E-01 | n.s. | n.s. |
|  | Validation | 3.2E-119 | 1.0E-03 | 1.3E-03 | 5.8E+02 | 1.1E+01 | 1.0E+01 | 1.3E-01 | n.s. | n.s. |
|  |  |  |  |  |  |  |  |  |  |  |
|  | **Model 4** | **RLE** | **EW** | **SIB** | **RLE** | **EW** | **SIB** | **RLE** | **EW** | **SIB** |
| Crystalized | Discovery | 1.9E-19 | 9.2E-13 | 1.6E-11 | 8.2E+01 | 5.1E+01 | 4.6E+01 | 2.1E-02 | 1.3E-02 | 1.2E-02 |
|  | Validation | 5.9E-19 | 4.1E-07 | 5.2E-09 | 8.0E+01 | 2.6E+01 | 3.4E+01 | 2.0E-02 | n.s. | 1.0E-02 |
| Fluid | Discovery | 2.9E-19 | 6.0E-08 | n.s. | 8.1E+01 | 2.9E+01 | 1.3E-01 | 2.1E-02 | n.s. | n.s. |
|  | Validation | 4.0E-22 | 9.1E-04 | n.s. | 9.5E+01 | 1.1E+01 | 1.5E-01 | 2.4E-02 | n.s. | n.s. |
| Total | Discovery | 5.6E-29 | 5.6E-15 | 4.1E-05 | 1.3E+02 | 6.2E+01 | 1.7E+01 | 3.2E-02 | 1.6E-02 | n.s. |
|  | Validation | 3.1E-30 | 2.7E-07 | 9.0E-05 | 1.3E+02 | 2.7E+01 | 1.5E+01 | 3.3E-02 | n.s. | n.s. |
|  |  |  |  |  |  |  |  |  |  |  |
|  | **Model 5** | SMA | EW | SIB | SMA | EW | SIB | SMA | EW | SIB |
| Crystalized | Discovery | 1.8E-38 | 1.8E-10 | 3.6E-10 | 1.7E+02 | 4.1E+01 | 4.0E+01 | 4.2E-02 | 1.0E-02 | 1.0E-02 |
|  | Validation | 1.2E-57 | 5.4E-05 | 2.7E-06 | 2.6E+02 | 1.6E+01 | 2.2E+01 | 6.4E-02 | n.s. | n.s. |
| Fluid | Discovery | 2.3E-27 | 8.8E-07 | n.s. | 1.2E+02 | 2.4E+01 | 4.5E-02 | 3.0E-02 | n.s. | n.s. |
|  | Validation | 4.2E-36 | 5.3E-03 | n.s. | 1.6E+02 | 7.8E+00 | 9.1E-01 | 4.0E-02 | 2.0E-03 | n.s. |
| Total | Discovery | 8.3E-49 | 1.7E-12 | 5.7E-04 | 2.2E+02 | 5.0E+01 | 1.2E+01 | 5.4E-02 | 1.3E-02 | n.s. |
|  | Validation | 2.0E-68 | 2.6E-05 | 1.8E-02 | 3.2E+02 | 1.8E+01 | 5.6E+00 | 7.6E-02 | n.s. | n.s. |

Table S3: Statistical significance for the main effect of family income (FI), and the residual effects of risk of lead exposure (RLE), excess weight (EW), siblings (SIB), screen media activity (SMA), parental education (PED), and area deprivation index (ADI) on fluid (FluidComp), crystalized (CrysComp), and total (CognComp) cognition composites.

|  | Discovery | | | Validation | | |
| --- | --- | --- | --- | --- | --- | --- |
|  | **Df** | **F value** | **P value** | **Df** | **F value** | **P value** |
| FluidComp | | | | | | |
| FI | 1 | 286.1 | < 2E-16 | 1 | 321.7 | < 2E-16 |
| RLE | 1 | 10.3 | 0.001 | 1 | 12.4 | 4E-04 |
| EW | 1 | 6.0 | 0.01 | 1 | 1.1 | n.s. |
| SIB | 1 | 0.3 | n.s. | 1 | 1.4 | n.s. |
| SMA | 1 | 32.9 | 1E-08 | 1 | 48.0 | 5E-12 |
| PED | 1 | 30.3 | 4E-04 | 1 | 13.5 | 2E-04 |
| ADI | 1 | 18.7 | 2E-05 | 1 | 4.2 | 0.04 |
| CrysComp | | | | | | |
| FI | 1 | 669.6 | < 2E-16 | 1 | 752.1 | < 2E-16 |
| RLE | 1 | 0.0 | n.s. | 1 | 0.5 | n.s. |
| EW | 1 | 9.1 | 0.003 | 1 | 3.0 | n.s. |
| SIB | 1 | 55.8 | 1E-13 | 1 | 55.7 | 1E-13 |
| SMA | 1 | 41.3 | 2E-10 | 1 | 73.5 | < 2E-16 |
| PED | 1 | 138.4 | < 2E-16 | 1 | 112.8 | < 2E-16 |
| ADI | 1 | 31.7 | 2E-08 | 1 | 25.0 | 6E-07 |
| CognComp | | | | | | |
| FI | 1 | 716.9 | < 2E-16 | 1 | 803.4 | < 2E-16 |
| RLE | 1 | 4.5 | 0.03 | 1 | 3.2 | n.s. |
| EW | 1 | 12.2 | 5E-04 | 1 | 2.9 | n.s. |
| SIB | 1 | 21.5 | 4E-06 | 1 | 29.2 | 7E-08 |
| SMA | 1 | 57.8 | 4E-14 | 1 | 91.6 | < 2E-16 |
| PED | 1 | 116.0 | < 2E-16 | 1 | 81.0 | < 2E-16 |
| ADI | 1 | 39.3 | 4E-10 | 1 | 20.0 | 8E-06 |

Factorial ANCOVA controlling for differences in scan manufacturer, intra scan head motion, intracranial volume, race, sex, and age which were used as covariates of no interest in the model. Residuals: 3873.

Table S4: Statistical significance corresponding to 5 different ANCOVA models associating total cortical volume and area, and average thickness with family income (FI; model 1), area deprivation index (ADI; model 2), parental education (PED; model3), risk of lead exposure (RLE; model 4), and screen media activity (SMA; model 5). Excess weight (EW) and siblings (SIB) were categorical factors of all 5 models

| Morphometric | Sample | P-value | | | F-value | | | Partial η^2^ | | |
| --- | --- | --- | --- | --- | --- | --- | --- | --- | --- | --- |
|  | **Model 1** | **FI** | **EW** | **SIB** | **FI** | **EW** | **SIB** | **FI** | **EW** | **SIB** |
| Volume | Discovery | 9.5E-259 | 1.8E-02 | n.s. | 1.4E+03 | 5.6E+00 | 8.9E-01 | 2.6E-01 | n.s. | n.s. |
|  | Validation | 2.0E-261 | 2.1E-13 | 2.2E-08 | 1.4E+03 | 5.4E+01 | 3.1E+01 | 2.6E-01 | 1.4E-02 | n.s. |
| Area | Discovery | 1.5E-187 | 6.6E-03 | 5.1E-11 | 9.5E+02 | 7.4E+00 | 4.3E+01 | 2.0E-01 | n.s. | 1.1E-02 |
|  | Validation | 3.1E-184 | n.s. | 1.0E-26 | 9.4E+02 | 1.2E+00 | 1.2E+02 | 1.9E-01 | n.s. | 2.9E-02 |
| Thickness | Discovery | 3.8E-23 | 2.5E-12 | 8.9E-11 | 9.9E+01 | 4.9E+01 | 4.2E+01 | 2.5E-02 | 1.3E-02 | 1.1E-02 |
|  | Validation | 2.8E-21 | 2.4E-16 | 1.2E-07 | 9.1E+01 | 6.8E+01 | 2.8E+01 | 2.3E-02 | 1.7E-02 | 1.0E-02 |
|  |  |  |  |  |  |  |  |  |  |  |
|  | **Model 2** | **ADI** | **EW** | **SIB** | **ADI** | **EW** | **SIB** | **ADI** | **EW** | **SIB** |
| Volume | Discovery | 6.3E-127 | 7.7E-09 | n.s. | 6.2E+02 | 3.3E+01 | 5.3E-02 | 1.4E-01 | 1.0E-02 | n.s. |
|  | Validation | 3.8E-152 | 7.7E-22 | 5.4E-05 | 7.6E+02 | 9.3E+01 | 1.6E+01 | 1.6E-01 | 2.4E-02 | n.s. |
| Area | Discovery | 2.5E-116 | n.s. | 1.1E-08 | 5.6E+02 | 1.2E-01 | 3.3E+01 | 1.3E-01 | n.s. | 1.0E-02 |
|  | Validation | 1.6E-115 | 4.4E-03 | 2.2E-21 | 5.6E+02 | 8.1E+00 | 9.1E+01 | 1.3E-01 | n.s. | 2.3E-02 |
| Thickness | Discovery | 3.0E-05 | 1.2E-16 | 6.1E-12 | 1.7E+01 | 6.9E+01 | 4.8E+01 | 4.5E-03 | 1.8E-02 | 1.2E-02 |
|  | Validation | 5.4E-10 | 7.9E-19 | 1.2E-08 | 3.9E+01 | 7.9E+01 | 3.3E+01 | 9.9E-03 | 2.0E-02 | 1.0E-02 |
|  |  |  |  |  |  |  |  |  |  |  |
|  | **Model 3** | **PED** | **EW** | **SIB** | **PED** | **EW** | **SIB** | **PED** | **EW** | **SIB** |
| Volume | Discovery | 3.0E-194 | 1.8E-04 | n.s. | 9.9E+02 | 1.4E+01 | 3.6E-01 | 2.0E-01 | n.s. | n.s. |
|  | Validation | 1.3E-167 | 1.9E-21 | 1.7E-03 | 8.4E+02 | 9.2E+01 | 9.8E+00 | 1.8E-01 | 2.3E-02 | n.s. |
| Area | Discovery | 6.4E-130 | n.s. | 1.8E-07 | 6.4E+02 | 1.9E+00 | 2.7E+01 | 1.4E-01 | n.s. | 1.0E-02 |
|  | Validation | 6.0E-114 | 2.9E-03 | 6.7E-18 | 5.5E+02 | 8.9E+00 | 7.5E+01 | 1.2E-01 | n.s. | 1.9E-02 |
| Thickness | Discovery | 2.6E-17 | 2.0E-13 | 8.4E-12 | 7.2E+01 | 5.4E+01 | 4.7E+01 | 1.8E-02 | 1.4E-02 | 1.2E-02 |
|  | Validation | 4.7E-13 | 2.3E-18 | 5.6E-09 | 5.3E+01 | 7.7E+01 | 3.4E+01 | 1.3E-02 | 2.0E-02 | 1.0E-02 |
|  |  |  |  |  |  |  |  |  |  |  |
|  | **Model 4** | **RLE** | **EW** | **SIB** | **RLE** | **EW** | **SIB** | **RLE** | **EW** | **SIB** |
| Volume | Discovery | 2.1E-63 | 2.2E-15 | n.s. | 2.9E+02 | 6.3E+01 | 4.8E-01 | 7.0E-02 | 1.6E-02 | n.s. |
|  | Validation | 3.5E-69 | 2.2E-30 | 3.8E-06 | 3.2E+02 | 1.3E+02 | 2.1E+01 | 7.7E-02 | 3.3E-02 | n.s. |
| Area | Discovery | 5.7E-33 | 2.5E-02 | 9.1E-10 | 1.5E+02 | 5.0E+00 | 3.8E+01 | 3.6E-02 | n.s. | 1.0E-02 |
|  | Validation | 3.7E-32 | 1.0E-06 | 1.6E-21 | 1.4E+02 | 2.4E+01 | 9.2E+01 | 3.5E-02 | n.s. | 2.3E-02 |
| Thickness | Discovery | 1.8E-13 | 6.2E-16 | 4.2E-10 | 5.5E+01 | 6.6E+01 | 3.9E+01 | 1.4E-02 | 1.7E-02 | 1.0E-02 |
|  | Validation | 9.1E-13 | 4.9E-19 | 2.2E-07 | 5.1E+01 | 8.0E+01 | 2.7E+01 | 1.3E-02 | 2.0E-02 | 1.0E-02 |
|  |  |  |  |  |  |  |  |  |  |  |
|  | **Model 5** | **SMA** | **EW** | **SIB** | **SMA** | **EW** | **SIB** | **SMA** | **EW** | **SIB** |
| Volume | Discovery | 6.9E-23 | 2.7E-17 | n.s. | 9.8E+01 | 7.2E+01 | 8.5E-01 | 2.5E-02 | 1.8E-02 | n.s. |
|  | Validation | 2.7E-45 | 2.8E-31 | 3.5E-02 | 2.0E+02 | 1.4E+02 | 4.4E+00 | 5.0E-02 | 3.4E-02 | n.s. |
| Area | Discovery | 1.6E-06 | 3.5E-03 | 1.4E-06 | 2.3E+01 | 8.5E+00 | 2.3E+01 | 5.9E-03 | n.s. | n.s. |
|  | Validation | 2.0E-34 | 3.6E-06 | 6.8E-15 | 1.5E+02 | 2.2E+01 | 6.1E+01 | 3.8E-02 | n.s. | 1.6E-02 |
| Thickness | Discovery | 5.0E-12 | 1.7E-15 | 1.6E-11 | 4.8E+01 | 6.4E+01 | 4.6E+01 | 1.2E-02 | 1.6E-02 | 1.2E-02 |
|  | Validation | 1.7E-04 | 2.4E-20 | 1.5E-09 | 1.4E+01 | 8.6E+01 | 3.7E+01 | 3.6E-03 | 2.2E-02 | n.s. |

Table S5: Statistical significance for the main effect of family income (FI), and the residual effects of risk of lead exposure (RLE), excess weight (EW), siblings (SIB), screen media activity (SMA), parental education (PED), area deprivation index (ADI) on whole-brain cortical volume and area and average cortical thickness.

|  | Discovery | | | Validation | | |
| --- | --- | --- | --- | --- | --- | --- |
|  | **Df** | **F value** | **P value** | **Df** | **F value** | **P value** |
| Cortical Volume | | | | | | |
| FI | 1 | 1383.8 | <2E-16 | 1 | 1398.3 | <2E-16 |
| RLE | 1 | 14.0 | 2E-04 | 1 | 20.0 | 8E-06 |
| EW | 1 | 2.6 | n.s. | 1 | 48.6 | 4E-12 |
| SIB | 1 | 1.5 | n.s. | 1 | 38.8 | 5E-10 |
| SMA | 1 | 1.5 | n.s. | 1 | 3.7 | n.s. |
| PED | 1 | 108.5 | <2E-16 | 1 | 61.6 | 6E-15 |
| ADI | 1 | 11.2 | 8E-04 | 1 | 0.3 | n.s. |
| Cortical Area | | | | | | |
| FI | 1 | 953.8 | <2E-16 | 1 | 936.1 | <2E-16 |
| RLE | 1 | 0.6 | n.s. | 1 | 0.5 | n.s. |
| EW | 1 | 10.1 | 0.001 | 1 | 1.0 | n.s. |
| SIB | 1 | 43.8 | 4E-11 | 1 | 120.4 | <2E-16 |
| SMA | 1 | 15.1 | 1E-04 | 1 | 7.8 | 0.005 |
| PED | 1 | 63.0 | 3E-15 | 1 | 38.9 | 5E-10 |
| ADI | 1 | 1.0 | n.s. | 1 | 0.5 | n.s. |
| Cortical Thickness | | | | | | |
| FI | 1 | 99.6 | <2E-16 | 1 | 90.8 | <2E-16 |
| RLE | 1 | 16.1 | 6E-05 | 1 | 15.5 | 8E-05 |
| EW | 1 | 42.3 | 9E-11 | 1 | 61.9 | 5E-15 |
| SIB | 1 | 38.7 | 5E-10 | 1 | 23.7 | 1E-06 |
| SMA | 1 | 9.3 | 0.002 | 1 | 0.1 | n.s. |
| PED | 1 | 4.1 | 0.04 | 1 | 2.5 | n.s. |
| ADI | 1 | 6.7 | 0.01 | 1 | 0.1 | n.s. |

Factorial ANCOVA controlling for differences in scan manufacturer, intra scan head motion, intracranial volume, race, sex, and age which were used as covariates of no interest in the model. Residuals: 3873.

Table S6: Statistical significance (P- and F- values, of main effects of family income (FI), excess weight (EW), and siblings (SIB) on cortical volume (CortVol), area (CortArea) and thickness (CortThick), averaged within cortical regions-of interest (ROI) defined in the Destrieux atlas, for ROIs that showed reproducible effects across the Discovery and Validation samples.

| **ROI** | **CortVol- FI** | | | **CortArea-FI** | | | **CortThick-FI** | | | **CortThick-EW** | | | **CortThick-SIB** | | |
| --- | --- | --- | --- | --- | --- | --- | --- | --- | --- | --- | --- | --- | --- | --- | --- |
|  | **P** | **F** | **slope %** | **P** | **F** | **slope %** | **P** | **F** | **slope %** | **P** | **F** | **L > O (CI) [mm]** | **P** | **F** | **Y > N (CI) [mm]** |
| **lh-G_and_S_cingul-Ant** | 2.76E-112 | 523 | 0.43(0.06) | 2.86E-102 | 474 | 0.42(0.07) | 2.90E-30 | 132 | 0.10(0.02) | 5.08E-03 | 8 | 0.03(0.03,0.04) | 5.08E-03 | 8 | 0.04(0.03,0.04) |
| **lh-G_and_S_cingul-Mid-Ant** | 7.94E-46 | 204 | 0.47(0.08) | 6.21E-28 | 121 | 0.37(0.08) | 5.69E-17 | 70 | 0.04(0.02) | 2.90E-30 | 132 | 0.03(0.02,0.03) | 2.90E-30 | 132 | 0.04(0.03,0.04) |
| **lh-G_and_S_cingul-Mid-Post** | 1.73E-54 | 245 | 0.34(0.07) | 3.23E-40 | 178 | 0.31(0.07) |  |  |  | 5.69E-17 | 70 | 0.02(0.01,0.02) |  |  |  |
| **lh-G_and_S_frontomargin** | 5.42E-92 | 424 | 0.48(0.07) | 3.12E-70 | 320 | 0.40(0.07) | 8.99E-20 | 83 | 0.10(0.03) | 8.99E-20 | 83 | 0.04(0.03,0.05) | 8.99E-20 | 83 | 0.04(0.03,0.05) |
| **lh-G_and_S_occipital_inf** | 9.60E-53 | 237 | 0.44(0.09) | 3.96E-31 | 136 | 0.34(0.08) | 1.92E-32 | 142 | 0.05(0.03) |  |  |  |  |  |  |
| **lh-G_and_S_paracentral** | 1.63E-43 | 194 | 0.32(0.08) | 1.47E-20 | 87 | 0.27(0.07) | 1.41E-32 | 142 | 0.06(0.03) |  |  |  | 1.41E-32 | 142 | 0.02(0.01,0.03) |
| **lh-G_and_S_subcentral** | 2.04E-88 | 407 | 0.56(0.08) | 2.57E-57 | 259 | 0.44(0.07) | 1.19E-29 | 129 | 0.13(0.03) |  |  |  | 1.19E-29 | 129 | 0.03(0.02,0.03) |
| **lh-G_and_S_transv_frontopol** | 5.31E-55 | 248 | 0.41(0.09) | 6.08E-42 | 186 | 0.39(0.09) |  |  |  | 1.06E-06 | 24 | 0.05(0.04,0.06) | 1.06E-06 | 24 | 0.03(0.02,0.04) |
| **lh-G_cingul-Post-dorsal** | 3.38E-58 | 263 | 0.50(0.10) | 2.13E-36 | 160 | 0.44(0.11) |  |  |  |  |  |  |  |  |  |
| **lh-G_cingul-Post-ventral** | 7.30E-11 | 43 | 0.27(0.16) | 1.78E-09 | 36 | 0.35(0.17) |  |  |  |  |  |  |  |  |  |
| **lh-G_cuneus** | 1.43E-72 | 331 | 0.35(0.08) | 3.74E-35 | 154 | 0.23(0.07) | 3.65E-34 | 150 | 0.09(0.03) |  |  |  |  |  |  |
| **lh-G_front_inf-Opercular** | 1.49E-52 | 236 | 0.36(0.07) | 1.07E-49 | 223 | 0.34(0.08) |  |  |  |  |  |  |  |  |  |
| **lh-G_front_inf-Orbital** | 4.06E-55 | 248 | 0.54(0.10) | 6.09E-44 | 196 | 0.46(0.10) |  |  |  |  |  |  |  |  |  |
| **lh-G_front_inf-Triangul** | 5.11E-14 | 57 | 0.28(0.09) | 3.61E-12 | 48 | 0.26(0.09) |  |  |  |  |  |  |  |  |  |
| **lh-G_front_middle** | 1.37E-104 | 485 | 0.50(0.07) | 4.22E-102 | 473 | 0.57(0.07) |  |  |  |  |  |  |  |  |  |
| **lh-G_front_sup** | 2.16E-163 | 776 | 0.36(0.05) | 1.71E-125 | 587 | 0.36(0.05) |  |  |  | 3.40E-05 | 17 | 0.03(0.02,0.04) | 3.40E-05 | 17 | 0.03(0.02,0.03) |
| **lh-G_Ins_lg_and_S_cent_ins** | 9.13E-42 | 185 | 0.43(0.08) | 8.99E-36 | 157 | 0.36(0.08) |  |  |  |  |  |  |  |  |  |
| **lh-G_insular_short** | 2.49E-68 | 311 | 0.31(0.06) | 4.99E-51 | 229 | 0.30(0.07) |  |  |  |  |  |  |  |  |  |
| **lh-G_oc-temp_lat-fusifor** | 1.49E-51 | 231 | 0.48(0.08) | 1.42E-31 | 138 | 0.45(0.08) | 1.07E-31 | 138 | 0.06(0.02) |  |  |  |  |  |  |
| **lh-G_oc-temp_med-Lingual** | 1.76E-56 | 255 | 0.4(0.08) | 1.38E-30 | 133 | 0.26(0.07) | 7.54E-32 | 139 | 0.11(0.03) |  |  |  |  |  |  |
| **lh-G_oc-temp_med-Parahip** | 3.24E-52 | 234 | 0.51(0.09) | 5.13E-23 | 98 | 0.39(0.09) | 9.28E-25 | 106 | 0.12(0.03) |  |  |  |  |  |  |
| **lh-G_occipital_middle** | 1.25E-109 | 510 | 0.56(0.08) | 7.56E-76 | 347 | 0.55(0.08) | 2.73E-21 | 90 | 0.02(0.03) |  |  |  | 2.73E-21 | 90 | 0.03(0.03,0.04) |
| **lh-G_occipital_sup** | 1.23E-79 | 365 | 0.35(0.08) | 5.45E-33 | 144 | 0.20(0.07) | 2.67E-43 | 193 | 0.08(0.04) |  |  |  | 2.67E-43 | 193 | 0.04(0.03,0.05) |
| **lh-G_orbital** | 1.05E-138 | 653 | 0.33(0.05) | 1.28E-103 | 480 | 0.28(0.05) | 5.80E-16 | 66 | 0.06(0.02) | 5.80E-16 | 66 | 0.03(0.02,0.04) | 5.80E-16 | 66 | 0.02(0.01,0.03) |
| **lh-G_pariet_inf-Angular** | 2.66E-24 | 104 | 0.30(0.07) | 1.25E-22 | 96 | 0.39(0.07) |  |  |  |  |  |  |  |  |  |
| **lh-G_pariet_inf-Supramar** | 7.96E-101 | 467 | 0.46(0.07) | 4.69E-69 | 314 | 0.44(0.08) | 1.57E-12 | 50 | 0.03(0.02) |  |  |  | 1.57E-12 | 50 | 0.03(0.03,0.04) |
| **lh-G_parietal_sup** | 7.39E-72 | 328 | 0.27(0.07) | 4.11E-63 | 286 | 0.32(0.07) |  |  |  |  |  |  |  |  |  |
| **lh-G_postcentral** | 5.33E-75 | 343 | 0.4(0.08) | 4.76E-49 | 220 | 0.36(0.07) | 2.02E-33 | 146 | 0.07(0.04) |  |  |  | 1.84E-08 | 32 | 0.03(0.02,0.04) |
| **lh-G_precentral** | 2.59E-103 | 479 | 0.32(0.06) | 1.38E-66 | 302 | 0.35(0.07) |  |  |  |  |  |  |  |  |  |
| **lh-G_precuneus** | 2.48E-89 | 411 | 0.35(0.06) | 2.47E-60 | 273 | 0.35(0.07) | 6.98E-10 | 38 | 0.02(0.02) |  |  |  |  |  |  |
| **lh-G_rectus** | 2.98E-30 | 132 | 0.25(0.06) | 2.11E-29 | 128 | 0.19(0.05) | 2.03E-10 | 41 | 0.08(0.03) |  |  |  |  |  |  |
| **lh-G_subcallosal** | 1.36E-08 | 32 | 0.41(0.21) | 7.84E-10 | 38 | 0.36(0.20) |  |  |  |  |  |  |  |  |  |
| **lh-G_temp_sup-G_T_transv** | 1.19E-29 | 129 | 0.35(0.09) |  |  |  |  |  |  |  |  |  |  |  |  |
| **lh-G_temp_sup-Lateral** | 1.65E-75 | 345 | 0.49(0.06) | 1.50E-47 | 212 | 0.35(0.06) | 6.28E-11 | 43 | 0.11(0.03) | 6.28E-11 | 43 | 0.03(0.02,0.04) | 6.28E-11 | 43 | 0.06(0.05,0.07) |
| **lh-G_temp_sup-Plan_polar** | 3.94E-45 | 201 | 0.38(0.09) | 1.44E-26 | 115 | 0.27(0.09) | 4.45E-10 | 39 | 0.11(0.03) |  |  |  |  |  |  |
| **lh-G_temp_sup-Plan_tempo** | 1.18E-35 | 157 | 0.53(0.10) | 3.41E-30 | 131 | 0.54(0.10) | 2.23E-08 | 31 | 0.02(0.03) |  |  |  |  |  |  |
| **lh-G_temporal_inf** | 1.52E-89 | 412 | 0.47(0.08) | 2.76E-74 | 339 | 0.42(0.08) | 2.11E-14 | 59 | 0.05(0.03) |  |  |  | 2.11E-14 | 59 | 0.03(0.02,0.03) |
| **lh-G_temporal_middle** | 2.48E-124 | 582 | 0.47(0.07) | 5.82E-77 | 352 | 0.39(0.07) | 2.46E-20 | 86 | 0.07(0.03) |  |  |  |  |  |  |
| **lh-Lat_Fis-ant-Horizont** | 2.86E-22 | 95 | 0.34(0.11) | 8.50E-24 | 102 | 0.32(0.09) | 3.77E-08 | 30 | 0.07(0.05) |  |  |  |  |  |  |
| **lh-Lat_Fis-ant-Vertical** | 2.45E-16 | 67 | 0.32(0.13) | 2.71E-16 | 67 | 0.28(0.13) |  |  |  |  |  |  |  |  |  |
| **lh-Lat_Fis-post** | 4.34E-71 | 324 | 0.56(0.08) | 1.37E-53 | 241 | 0.47(0.08) | 1.16E-35 | 157 | 0.08(0.02) |  |  |  |  |  |  |
| **lh-Pole_occipital** | 7.60E-50 | 223 | 0.33(0.07) | 3.31E-14 | 58 | 0.25(0.06) | 3.24E-38 | 169 | 0.05(0.04) |  |  |  | 3.24E-38 | 169 | 0.05(0.05,0.06) |
| **lh-Pole_temporal** | 4.56E-84 | 386 | 0.33(0.06) | 1.15E-76 | 351 | 0.29(0.06) |  |  |  | 2.46E-03 | 9 | 0.03(0.02,0.04) |  |  |  |
| **lh-S_calcarine** | 4.96E-63 | 286 | 0.31(0.08) | 1.11E-33 | 148 | 0.26(0.07) | 1.85E-38 | 170 | 0.04(0.03) | 1.85E-38 | 170 | 0.02(0.02,0.03) |  |  |  |
| **lh-S_central** | 4.01E-92 | 425 | 0.44(0.07) | 4.28E-79 | 362 | 0.36(0.05) | 1.99E-46 | 207 | 0.09(0.03) |  |  |  |  |  |  |
| **lh-S_cingul-Marginalis** | 2.97E-39 | 174 | 0.40(0.08) | 1.02E-35 | 157 | 0.35(0.07) | 1.98E-22 | 95 | 0.07(0.03) |  |  |  |  |  |  |
| **lh-S_circular_insula_ant** | 2.00E-50 | 226 | 0.34(0.08) | 1.65E-29 | 128 | 0.27(0.08) | 3.24E-23 | 99 | 0.06(0.03) | 3.24E-23 | 99 | 0.02(0.02,0.03) |  |  |  |
| **lh-S_circular_insula_inf** | 1.02E-26 | 115 | 0.31(0.07) | 1.73E-22 | 96 | 0.23(0.05) | 4.31E-19 | 80 | 0.09(0.03) |  |  |  |  |  |  |
| **lh-S_circular_insula_sup** | 2.02E-89 | 412 | 0.35(0.05) | 2.72E-73 | 334 | 0.30(0.05) | 2.64E-32 | 141 | 0.06(0.02) |  |  |  |  |  |  |
| **lh-S_collat_transv_ant** | 7.30E-77 | 351 | 0.71(0.10) | 2.10E-60 | 273 | 0.71(0.11) |  |  |  |  |  |  |  |  |  |
| **lh-S_collat_transv_post** | 4.36E-34 | 149 | 0.41(0.11) | 4.10E-40 | 178 | 0.44(0.10) |  |  |  |  |  |  |  |  |  |
| **lh-S_front_inf** | 1.66E-29 | 128 | 0.42(0.09) | 1.72E-27 | 119 | 0.36(0.08) | 4.96E-23 | 98 | 0.08(0.02) | 4.96E-23 | 98 | 0.02(0.02,0.03) |  |  |  |
| **lh-S_front_middle** | 1.09E-31 | 138 | 0.48(0.10) | 2.39E-35 | 155 | 0.52(0.09) | 1.89E-12 | 50 | 0.04(0.03) | 1.89E-12 | 50 | 0.03(0.02,0.03) |  |  |  |
| **lh-S_front_sup** | 3.15E-62 | 282 | 0.57(0.07) | 9.61E-68 | 308 | 0.51(0.07) | 1.65E-22 | 96 | 0.10(0.02) |  |  |  |  |  |  |
| **lh-S_intrapariet_and_P_trans** | 4.76E-31 | 135 | 0.24(0.07) | 7.12E-22 | 93 | 0.25(0.07) | 2.27E-25 | 109 | 0.01(0.02) |  |  |  |  |  |  |
| **lh-S_oc-temp_lat** | 2.39E-52 | 235 | 0.55(0.10) | 1.15E-38 | 171 | 0.53(0.10) | 5.80E-14 | 57 | 0.02(0.03) |  |  |  |  |  |  |
| **lh-S_oc-temp_med_and_Lingual** | 2.13E-52 | 235 | 0.38(0.07) | 2.28E-20 | 86 | 0.27(0.07) | 3.27E-62 | 282 | 0.10(0.02) |  |  |  |  |  |  |
| **lh-S_oc_middle_and_Lunatus** | 4.62E-61 | 276 | 0.63(0.12) | 6.32E-43 | 191 | 0.64(0.11) | 3.70E-29 | 127 | -0.04(0.03) | 3.70E-29 | 127 | 0.03(0.02,0.03) |  |  |  |
| **lh-S_oc_sup_and_transversal** | 2.32E-23 | 100 | 0.09(0.09) | 7.07E-12 | 47 | 0.09(0.08) | 1.90E-28 | 123 | 0.01(0.03) | 1.90E-28 | 123 | 0.03(0.02,0.03) |  |  |  |
| **lh-S_occipital_ant** | 3.11E-16 | 67 | 0.48(0.13) | 8.42E-10 | 38 | 0.47(0.12) | 3.60E-15 | 62 | 0.01(0.03) |  |  |  |  |  |  |
| **lh-S_orbital-H_Shaped** | 9.43E-96 | 442 | 0.37(0.06) | 1.54E-95 | 441 | 0.33(0.06) | 5.54E-12 | 48 | 0.07(0.03) | 5.54E-12 | 48 | 0.03(0.02,0.04) | 5.54E-12 | 48 | 0.05(0.04,0.06) |
| **lh-S_orbital_lateral** | 2.03E-23 | 100 | 0.44(0.10) | 7.14E-19 | 79 | 0.46(0.10) | 1.97E-08 | 32 | 0.06(0.05) |  |  |  |  |  |  |
| **lh-S_parieto_occipital** | 2.53E-55 | 249 | 0.42(0.08) | 1.41E-31 | 138 | 0.34(0.08) | 6.27E-45 | 200 | 0.07(0.03) |  |  |  |  |  |  |
| **lh-S_pericallosal** | 1.93E-28 | 123 | 0.40(0.11) | 1.91E-64 | 292 | 0.55(0.08) |  |  |  |  |  |  |  |  |  |
| **lh-S_postcentral** | 3.84E-54 | 244 | 0.39(0.08) | 4.30E-37 | 163 | 0.30(0.07) | 2.89E-45 | 202 | 0.10(0.02) |  |  |  |  |  |  |
| **lh-S_precentral-inf-part** | 1.54E-69 | 317 | 0.56(0.09) | 1.45E-65 | 298 | 0.50(0.08) | 4.81E-28 | 121 | 0.10(0.02) |  |  |  |  |  |  |
| **lh-S_precentral-sup-part** | 2.94E-14 | 58 | 0.44(0.10) | 1.42E-12 | 50 | 0.39(0.10) | 1.42E-15 | 64 | 0.11(0.03) |  |  |  |  |  |  |
| **lh-S_suborbital** |  |  |  | 1.77E-16 | 68 | 0.24(0.08) |  |  |  |  |  |  |  |  |  |
| **lh-S_subparietal** | 9.20E-36 | 157 | 0.43(0.11) | 1.73E-30 | 133 | 0.40(0.10) | 6.81E-16 | 65 | 0.01(0.03) | 6.81E-16 | 65 | 0.02(0.02,0.03) | 6.81E-16 | 65 | 0.02(0.02,0.03) |
| **lh-S_temporal_inf** | 3.61E-31 | 136 | 0.29(0.11) | 7.40E-22 | 93 | 0.26(0.10) | 1.52E-12 | 50 | 0.03(0.03) |  |  |  |  |  |  |
| **lh-S_temporal_sup** | 8.86E-61 | 275 | 0.36(0.06) | 8.45E-46 | 204 | 0.34(0.06) | 3.66E-20 | 85 | 0.01(0.02) | 3.66E-20 | 85 | 0.02(0.01,0.02) |  |  |  |
| **lh-S_temporal_transverse** | 1.12E-12 | 51 | 0.37(0.10) | 2.99E-23 | 99 | 0.43(0.09) |  |  |  |  |  |  |  |  |  |
| **rh-G_and_S_cingul-Ant** | 9.23E-101 | 467 | 0.39(0.06) | 3.24E-114 | 532 | 0.40(0.06) |  |  |  | 2.00E-04 | 14 | 0.04(0.03,0.05) |  |  |  |
| **rh-G_and_S_cingul-Mid-Ant** | 2.59E-70 | 320 | 0.40(0.07) | 1.79E-47 | 212 | 0.34(0.07) | 1.59E-22 | 96 | 0.07(0.02) | 1.59E-22 | 96 | 0.02(0.01,0.03) | 1.59E-22 | 96 | 0.02(0.01,0.03) |
| **rh-G_and_S_cingul-Mid-Post** | 2.99E-66 | 301 | 0.42(0.07) | 9.82E-56 | 251 | 0.40(0.07) | 3.65E-12 | 48 | 0.04(0.02) |  |  |  |  |  |  |
| **rh-G_and_S_frontomargin** | 1.08E-89 | 413 | 0.47(0.07) | 1.03E-74 | 341 | 0.43(0.07) | 1.14E-18 | 78 | 0.06(0.04) | 1.14E-18 | 78 | 0.06(0.05,0.07) |  |  |  |
| **rh-G_and_S_occipital_inf** | 2.66E-52 | 235 | 0.42(0.09) | 4.08E-34 | 150 | 0.37(0.09) | 9.59E-25 | 106 | 0.05(0.03) |  |  |  |  |  |  |
| **rh-G_and_S_paracentral** | 2.16E-28 | 123 | 0.24(0.08) | 1.15E-11 | 46 | 0.19(0.07) | 1.51E-29 | 128 | 0.07(0.03) |  |  |  |  |  |  |
| **rh-G_and_S_subcentral** | 2.22E-72 | 330 | 0.51(0.08) | 7.41E-50 | 223 | 0.43(0.08) | 2.97E-16 | 67 | 0.08(0.03) |  |  |  |  |  |  |
| **rh-G_and_S_transv_frontopol** | 1.80E-60 | 273 | 0.34(0.08) | 2.37E-50 | 226 | 0.40(0.09) |  |  |  | 2.65E-04 | 13 | 0.06(0.05,0.07) | 2.65E-04 | 13 | 0.03(0.02,0.05) |
| **rh-G_cingul-Post-dorsal** | 6.61E-40 | 177 | 0.44(0.10) | 8.37E-27 | 116 | 0.41(0.10) |  |  |  |  |  |  |  |  |  |
| **rh-G_cuneus** | 5.54E-63 | 285 | 0.36(0.08) | 2.71E-27 | 118 | 0.23(0.07) | 1.29E-44 | 199 | 0.11(0.03) |  |  |  |  |  |  |
| **rh-G_front_inf-Opercular** | 3.15E-44 | 197 | 0.31(0.08) | 1.46E-40 | 180 | 0.28(0.08) |  |  |  |  |  |  |  |  |  |
| **rh-G_front_inf-Orbital** |  |  |  | 1.17E-09 | 37 | 0.24(0.10) |  |  |  | 7.75E-03 | 7 | 0.03(0.02,0.04) |  |  |  |
| **rh-G_front_middle** | 5.28E-83 | 381 | 0.45(0.07) | 1.08E-92 | 428 | 0.54(0.07) |  |  |  |  |  |  |  |  |  |
| **rh-G_front_sup** | 1.58E-174 | 832 | 0.43(0.05) | 1.20E-148 | 702 | 0.45(0.06) |  |  |  | 5.34E-03 | 8 | 0.03(0.02,0.04) |  |  |  |
| **rh-G_Ins_lg_and_S_cent_ins** | 5.02E-61 | 276 | 0.50(0.08) | 1.57E-45 | 203 | 0.40(0.08) |  |  |  |  |  |  |  |  |  |
| **rh-G_insular_short** | 2.78E-53 | 239 | 0.31(0.07) | 4.28E-40 | 178 | 0.33(0.08) |  |  |  |  |  |  |  |  |  |
| **rh-G_oc-temp_lat-fusifor** | 1.88E-44 | 198 | 0.54(0.09) | 1.46E-22 | 96 | 0.46(0.09) | 2.05E-46 | 207 | 0.09(0.02) | 2.05E-46 | 207 | 0.02(0.02,0.03) | 2.05E-46 | 207 | 0.03(0.02,0.04) |
| **rh-G_oc-temp_med-Lingual** | 2.83E-97 | 450 | 0.47(0.07) | 1.82E-64 | 292 | 0.37(0.06) | 4.27E-30 | 131 | 0.09(0.03) |  |  |  |  |  |  |
| **rh-G_oc-temp_med-Parahip** | 4.26E-52 | 234 | 0.62(0.09) | 3.21E-27 | 118 | 0.54(0.09) | 9.98E-14 | 56 | 0.08(0.03) |  |  |  |  |  |  |
| **rh-G_occipital_middle** | 2.93E-93 | 430 | 0.50(0.08) | 4.99E-58 | 262 | 0.45(0.08) | 3.91E-19 | 80 | 0.02(0.03) |  |  |  | 3.91E-19 | 80 | 0.03(0.02,0.04) |
| **rh-G_occipital_sup** | 8.55E-92 | 423 | 0.46(0.08) | 9.81E-38 | 166 | 0.27(0.07) | 7.81E-51 | 228 | 0.13(0.03) |  |  |  | 7.81E-51 | 228 | 0.04(0.03,0.05) |
| **rh-G_orbital** | 2.46E-146 | 690 | 0.37(0.05) | 5.00E-112 | 521 | 0.34(0.05) | 7.71E-15 | 61 | 0.04(0.02) | 7.71E-15 | 61 | 0.03(0.03,0.04) |  |  |  |
| **rh-G_pariet_inf-Angular** | 1.55E-34 | 152 | 0.29(0.07) | 2.39E-29 | 127 | 0.37(0.07) |  |  |  |  |  |  |  |  |  |
| **rh-G_pariet_inf-Supramar** | 6.96E-67 | 304 | 0.47(0.08) | 1.70E-49 | 222 | 0.46(0.08) | 7.21E-10 | 38 | 0.01(0.02) |  |  |  | 7.21E-10 | 38 | 0.04(0.04,0.05) |
| **rh-G_parietal_sup** | 3.63E-52 | 234 | 0.21(0.08) | 6.57E-51 | 228 | 0.29(0.08) |  |  |  |  |  |  |  |  |  |
| **rh-G_postcentral** | 8.26E-46 | 204 | 0.26(0.08) | 4.39E-30 | 131 | 0.24(0.07) | 1.26E-18 | 78 | 0.03(0.04) |  |  |  |  |  |  |
| **rh-G_precentral** | 8.06E-105 | 486 | 0.37(0.06) | 3.23E-59 | 268 | 0.38(0.07) |  |  |  |  |  |  | 4.22E-08 | 30 | 0.03(0.02,0.04) |
| **rh-G_precuneus** | 1.34E-88 | 408 | 0.32(0.06) | 2.09E-71 | 325 | 0.38(0.07) |  |  |  |  |  |  |  |  |  |
| **rh-G_rectus** | 2.15E-21 | 91 | 0.26(0.07) | 2.93E-26 | 113 | 0.24(0.06) |  |  |  |  |  |  |  |  |  |
| **rh-G_temp_sup-G_T_transv** | 5.48E-44 | 196 | 0.48(0.09) | 1.45E-24 | 105 | 0.40(0.08) | 1.08E-15 | 65 | 0.05(0.03) |  |  |  |  |  |  |
| **rh-G_temp_sup-Lateral** | 4.13E-92 | 425 | 0.47(0.06) | 7.19E-59 | 266 | 0.41(0.06) | 4.82E-19 | 80 | 0.08(0.03) |  |  |  | 4.82E-19 | 80 | 0.06(0.05,0.07) |
| **rh-G_temp_sup-Plan_polar** | 1.70E-38 | 170 | 0.40(0.08) | 9.25E-25 | 106 | 0.35(0.08) |  |  |  |  |  |  |  |  |  |
| **rh-G_temp_sup-Plan_tempo** | 3.02E-29 | 127 | 0.50(0.09) | 6.59E-19 | 79 | 0.48(0.08) | 3.49E-17 | 71 | 0.06(0.03) |  |  |  |  |  |  |
| **rh-G_temporal_inf** | 1.05E-87 | 404 | 0.41(0.08) | 5.81E-65 | 295 | 0.36(0.07) | 1.26E-18 | 78 | 0.05(0.02) |  |  |  | 1.26E-18 | 78 | 0.03(0.02,0.04) |
| **rh-G_temporal_middle** | 1.40E-176 | 842 | 0.48(0.06) | 2.45E-104 | 484 | 0.43(0.06) | 1.04E-23 | 101 | 0.04(0.02) | 1.04E-23 | 101 | 0.03(0.02,0.03) | 1.04E-23 | 101 | 0.03(0.02,0.04) |
| **rh-Lat_Fis-ant-Horizont** | 1.49E-10 | 41 | 0.29(0.11) | 1.72E-13 | 54 | 0.27(0.09) |  |  |  | 1.24E-04 | 15 | 0.03(0.02,0.04) |  |  |  |
| **rh-Lat_Fis-ant-Vertical** | 6.79E-10 | 38 | 0.29(0.14) | 2.71E-11 | 44 | 0.30(0.14) |  |  |  |  |  |  |  |  |  |
| **rh-Lat_Fis-post** | 4.74E-32 | 140 | 0.27(0.06) | 1.46E-18 | 78 | 0.24(0.05) | 2.51E-39 | 174 | 0.05(0.02) | 2.51E-39 | 174 | 0.02(0.02,0.03) |  |  |  |
| **rh-Pole_occipital** | 1.08E-63 | 289 | 0.32(0.07) | 4.33E-14 | 57 | 0.21(0.06) | 1.98E-48 | 217 | 0.07(0.03) |  |  |  |  |  |  |
| **rh-Pole_temporal** | 2.34E-60 | 273 | 0.24(0.05) | 1.07E-75 | 346 | 0.30(0.06) |  |  |  |  |  |  | 1.98E-48 | 217 | 0.05(0.04,0.05) |
| **rh-S_calcarine** | 7.46E-67 | 304 | 0.39(0.08) | 2.95E-39 | 174 | 0.32(0.07) | 1.80E-56 | 255 | 0.10(0.03) | 1.80E-56 | 255 | 0.02(0.02,0.03) |  |  |  |
| **rh-S_central** | 4.93E-55 | 248 | 0.38(0.07) | 6.80E-48 | 214 | 0.30(0.05) | 2.54E-30 | 132 | 0.09(0.03) |  |  |  |  |  |  |
| **rh-S_cingul-Marginalis** | 3.08E-23 | 99 | 0.28(0.09) | 4.60E-25 | 108 | 0.30(0.08) | 3.01E-16 | 67 | 0.03(0.02) |  |  |  |  |  |  |
| **rh-S_circular_insula_ant** | 5.80E-30 | 130 | 0.28(0.08) | 2.91E-12 | 49 | 0.22(0.08) | 4.74E-23 | 98 | 0.06(0.03) | 4.74E-23 | 98 | 0.03(0.02,0.04) |  |  |  |
| **rh-S_circular_insula_inf** | 2.41E-36 | 160 | 0.34(0.07) | 2.36E-32 | 141 | 0.28(0.06) | 4.74E-19 | 80 | 0.07(0.03) |  |  |  |  |  |  |
| **rh-S_circular_insula_sup** | 8.40E-44 | 195 | 0.29(0.06) | 3.28E-39 | 173 | 0.27(0.06) | 2.08E-22 | 95 | 0.03(0.02) | 2.08E-22 | 95 | 0.02(0.02,0.03) |  |  |  |
| **rh-S_collat_transv_ant** | 1.16E-78 | 360 | 0.63(0.10) | 6.46E-62 | 280 | 0.66(0.10) |  |  |  |  |  |  |  |  |  |
| **rh-S_collat_transv_post** | 3.89E-27 | 117 | 0.35(0.13) | 9.94E-21 | 88 | 0.32(0.11) | 2.08E-20 | 86 | 0.07(0.04) |  |  |  |  |  |  |
| **rh-S_front_inf** | 5.08E-34 | 149 | 0.39(0.08) | 4.62E-37 | 163 | 0.40(0.07) | 1.71E-18 | 77 | 0.05(0.02) | 1.71E-18 | 77 | 0.03(0.03,0.04) | 1.71E-18 | 77 | 0.02(0.01,0.02) |
| **rh-S_front_middle** | 1.28E-26 | 115 | 0.41(0.09) | 7.30E-29 | 125 | 0.40(0.08) | 2.95E-19 | 81 | 0.07(0.03) | 2.95E-19 | 81 | 0.04(0.04,0.05) |  |  |  |
| **rh-S_front_sup** | 1.58E-57 | 260 | 0.52(0.08) | 1.28E-69 | 317 | 0.53(0.07) | 3.62E-11 | 44 | 0.05(0.02) |  |  |  |  |  |  |
| **rh-S_intrapariet_and_P_trans** | 4.93E-31 | 135 | 0.20(0.07) | 1.66E-26 | 114 | 0.23(0.07) | 5.41E-11 | 43 | -0.03(0.02) |  |  |  |  |  |  |
| **rh-S_oc-temp_lat** | 4.46E-51 | 229 | 0.47(0.10) | 1.16E-29 | 129 | 0.44(0.10) | 2.09E-26 | 114 | 0.06(0.03) |  |  |  | 2.09E-26 | 114 | 0.02(0.02,0.03) |
| **rh-S_oc-temp_med_and_Lingual** | 3.09E-63 | 287 | 0.38(0.07) | 3.74E-28 | 122 | 0.30(0.07) | 1.28E-60 | 274 | 0.09(0.02) |  |  |  |  |  |  |
| **rh-S_oc_middle_and_Lunatus** | 1.16E-53 | 241 | 0.49(0.13) | 1.91E-35 | 156 | 0.44(0.12) | 2.02E-31 | 137 | 0.04(0.04) |  |  |  | 2.02E-31 | 137 | 0.03(0.02,0.03) |
| **rh-S_oc_sup_and_transversal** | 2.04E-25 | 109 | 0.26(0.09) | 1.13E-14 | 60 | 0.25(0.08) | 1.19E-21 | 92 | 0.01(0.03) |  |  |  |  |  |  |
| **rh-S_occipital_ant** | 4.16E-34 | 150 | 0.58(0.13) | 1.25E-19 | 83 | 0.55(0.12) | 1.69E-27 | 119 | 0.04(0.03) |  |  |  |  |  |  |
| **rh-S_orbital-H_Shaped** | 3.06E-94 | 435 | 0.41(0.06) | 1.12E-102 | 476 | 0.40(0.06) | 1.49E-11 | 46 | 0.05(0.05) | 5.70E-09 | 34 | 0.04(0.03,0.04) |  |  |  |
| **rh-S_orbital_lateral** |  |  |  |  |  |  |  |  |  | 1.49E-11 | 46 | 0.04(0.03,0.05) |  |  |  |
| **rh-S_orbital_med-olfact** |  |  |  |  |  |  |  |  |  | 6.43E-01 | 0 | 0.03(0.02,0.04) |  |  |  |
| **rh-S_parieto_occipital** | 2.50E-56 | 254 | 0.44(0.09) | 1.32E-36 | 161 | 0.35(0.08) | 6.87E-38 | 167 | 0.10(0.03) |  |  |  |  |  |  |
| **rh-S_pericallosal** |  |  |  | 7.24E-30 | 130 | 0.31(0.08) | 4.22E-12 | 48 | -0.22(0.05) |  |  |  |  |  |  |
| **rh-S_postcentral** | 2.65E-34 | 150 | 0.41(0.10) | 2.74E-29 | 127 | 0.36(0.09) | 3.83E-22 | 94 | 0.04(0.03) |  |  |  |  |  |  |
| **rh-S_precentral-inf-part** | 1.23E-70 | 322 | 0.58(0.09) | 2.48E-73 | 335 | 0.53(0.08) | 3.93E-16 | 67 | 0.07(0.02) | 3.93E-16 | 67 | 0.02(0.01,0.02) |  |  |  |
| **rh-S_precentral-sup-part** | 2.81E-21 | 90 | 0.51(0.11) | 5.17E-22 | 94 | 0.50(0.10) |  |  |  |  |  |  |  |  |  |
| **rh-S_subparietal** | 2.00E-15 | 63 | 0.38(0.12) | 8.06E-14 | 56 | 0.36(0.12) |  |  |  |  |  |  | 2.70E-05 | 18 | 0.02(0.02,0.03) |
| **rh-S_temporal_inf** | 1.65E-54 | 245 | 0.50(0.10) | 1.71E-45 | 203 | 0.51(0.10) | 3.18E-16 | 67 | 0.03(0.02) |  |  |  |  |  |  |
| **rh-S_temporal_sup** | 2.34E-108 | 504 | 0.45(0.06) | 2.05E-83 | 383 | 0.41(0.06) | 8.40E-42 | 186 | 0.04(0.02) |  |  |  |  |  |  |
| **rh-S_temporal_transverse** | 1.41E-22 | 96 | 0.35(0.10) | 1.32E-17 | 73 | 0.44(0.10) | 2.90E-08 | 31 | 0.01(0.05) |  |  |  |  |  |  |

Factorial ANOVA included main effects of FI, EW, SIB, risk of lead exposure (RLE), area deprivation index (ADI), screen media activity, parental education, sex, and age on brain morphometrics (cortical area, thickness and volume), while controlling for differences in scan manufacturer, intra scan head motion, intracranial volume and race which were used as covariates of no interest in the model. Effects survive Bonferroni corrections for 148 comparisons. Tukey's honestly significance test was used to assess group differences and confidence intervals (CI). A linear model was used to estimate relative slopes and their corresponding errors (values in parentheses).

**References**

1. Luciana M, Bjork J, Nagel B, Barch D, Gonzalez R, Nixon S *et al.* Adolescent neurocognitive development and impacts of substance use: Overview of the adolescent brain cognitive development (ABCD) baseline neurocognition battery. *Dev Cogn Neurosci* 2018; **32:** 67-79.
